# Nanotheranostics through Mitochondria-targeted Delivery with Fluorescent Peptidomimetic Nanohybrids for Apoptosis Induction of Brain Cancer Cells

**DOI:** 10.7150/ntno.54491

**Published:** 2021-02-08

**Authors:** Isadora C. Carvalho, Alexandra A. P. Mansur, Sandhra M. Carvalho, Herman S. Mansur

**Affiliations:** Center of Nanoscience, Nanotechnology, and Innovation - CeNano2I, Department of Metallurgical and Materials Engineering, Federal University of Minas Gerais - UFMG, Av. Antônio Carlos, 6627 - Belo Horizonte/MG, Brazil.

**Keywords:** nanoconjugates, fluorescent nanoparticles, dual-functional bioconjugates, cancer nanomedicine, dual-targeted drug delivery

## Abstract

**Overview:** Malignant brain tumors remain one of the greatest challenges faced by health professionals and scientists among the utmost lethal forms of cancer. Nanotheranostics can play a pivotal role in developing revolutionary nanoarchitectures with multifunctional and multimodal capabilities to fight cancer. Mitochondria are vital organelles to eukaryotic cells, which have been recognized as a significant target in cancer therapy where, by damaging the mitochondria, it will cause irreparable cell death or apoptosis.

**Methods:** We designed and produced novel hybrid nanostructures comprising a fluorescent semiconductor core (AgInS_2_, AIS) and cysteine-modified carboxymethylcellulose (termed thiomer, CMC_Cys) conjugated with mitochondria-targeting peptides (KLA) forming a macromolecular shell for combining bioimaging and for inducing brain cancer cell (U-87 MG) death.

**Results:** The optical and physicochemical properties of the nanoconjugates demonstrated suitability as photoluminescent nanostructures for cell bioimaging and intracellular tracking. Additionally, the results proved a remarkable killing activity towards glioblastoma cells of cysteine-bearing CMC conjugates coupled with KLA peptides through the half-maximal effective concentration values, approximately 70-fold higher compared to the conjugate analogs without Cys residues. Moreover, these thiomer-based pro-apoptotic drug nanoconjugates displayed higher lethality against U-87 MG cancer cells than doxorubicin, a model drug in chemotherapy, although extremely toxic. Remarkably, these peptidomimetic nanohybrids demonstrated a relative “protective effect” regarding healthy cells while maintaining high killing activity towards malignant brain cells.

**Conclusion:** These findings pave the way for developing hybrid nanoarchitectures applied as targeted multifunctional platforms for simultaneous imaging and therapy against cancer while minimizing the high systemic toxicity and side-effects of conventional drugs in anticancer chemotherapy.

## Introduction

The continuous fight against cancer, the second leading cause of death worldwide, has been favoring multidisciplinary approaches for producing robust diagnosis and therapy alternatives (referred to as theranostic) to the currently applied conventional chemotherapy. Brain tumors are among the most feared of all forms of cancer, where the vast majority of adults diagnosed with glioblastoma (referred to as GBM) have an adverse prognosis. GBM is the most life-threatening and aggressive primary brain cancer and remains predominantly incurable, with a median survival of only 15 months. This scenario is aggravated because, despite the current development in mainstream GBM therapy, the complete remission for glioma seems to be out of reach, and regrettably, only very few treatment possibilities are offered 1,2*.* Moreover, conventional treatments relying on toxic drugs often cause numerous side-effects to patients, with narrow therapeutic indexes, and leading to multidrug-resistance of brain tumors, which constitute major challenges for developing novel therapies for most malignancies, including GBM 3-5. Therefore, an essential approach for overcoming this crucial obstacle is the development of targeted and optimized delivery systems. Fortunately, advancements in nanotechnology have been enabling new multimodal therapy approaches, such as the use of nanoparticles and hybrids in drug delivery systems, as well as the progress of synthetic therapeutic peptides. Peptides present themselves as promisor components of molecular devices and have been gaining attention as candidates for the next generation of anticancer therapeutics 6,7. Offering advantages such as fast synthesis and functionalization, apoptosis-inducing peptides are also less immunogenic compared to recombinant antibodies or protein alternatives 6,7. Furthermore, they can better penetrate the tissues and oncogenic regions due to their smaller size 8. Owing to their resemblance to protein structure, peptides present greater efficacy, selectivity, and specificity when paralleled to other small organic molecules used as chemotherapeutic agents 8. They can also be degraded by proteases and generate products with lower chances of developing systemic toxicity and tissue accumulation 9. Therefore, anticancer peptides display a broad scope and present distinct activities against abnormalities that appear to be similar in some types of cancer 10. The therapeutic activity of peptides is often allocated to their structural properties such as net charge, amphipathicity, and secondary structure in the membrane 11. Amino acids are essential units (building blocks) of peptides and also the base of proteome and, therefore, changes in single residues can completely alter the function of active peptide sequences 12. More recently, peptides and structures bearing L-cysteine (Cys) residues are gaining crescent interest owing to the multiple roles played in the complex mechanisms and metabolic pathways of biological systems, comprising functions such as redox reactions, signaling, and bioadhesion 13,14. Moreover, thiol groups of Cys residues can interact with exofacial thiol groups that exist in many proteins (*e.g.,* integrins) that are overexpressed by cancer cells and significantly influence cell-uptake 15-17, which has been exploited for drug delivery of therapeutic entities 18. However, although peptide therapeutics have great potentials in the treatment of many diseases including cancer, the main drawback is their short lifetime *in vivo* due to rapid degradation by enzymes, low stability in plasma, and rapid clearance from circulation 19. Therefore, the introduction of synthetic scaffolds and macromolecules decorated with peptides or peptide fragments can overcome many of these problems by improving metabolic stability and pharmacokinetics 19. To this end, a new field of research has emerged termed peptidomimetic nanosystems, which essentially encompasses the process of developing nanostructures from macromolecular chains by mimicking the pattern of hydrogen bonding, weak non-polar, polar, and electrostatic interactions found in peptides. Thus, innovative strategies based on peptidomimetic hybrids comprising polymer-peptide drug conjugates come as attractive options of combining unique biological properties of peptides as well as desired bioengineered physiochemical properties of polymer macromolecules 19-21.

To this end, polysaccharides have been of great interest in designing advanced functionalized macromolecular structures owing to their structural characteristics from natural biological sources, which often render them suitable biocompatibility. Additionally, they can be easily modified with biochemical functionalities, which provide innumerous opportunities for biomedical applications. Semi-natural polysaccharides, such as carboxymethylcellulose (CMC), have been chemically modified to be applied in drug carrier and delivery, and gene therapy. Besides, polysaccharide derivatives bearing thiol functional groups (generally named as thiomers) such as cysteine and cysteine-rich peptides have been studied for multiple biological-related functions, including enhancing mucoadhesion and for promoting cell interactions at biointerfaces. Nevertheless, the delivery of specific drugs to the cell and subcellular organelles is one of the major problems that remain to be solved 17,22-24.

In this scenario, subcellular drug targeting strategies present a vast realm to be explored with great potential to overcome significant drawbacks and limitations related to drug toxicity experimented in currently applied cancer chemotherapy, as recently reported in an elegant review published by Gao *et al.* 25. Thus, widely known as the cell machinery, the mitochondrion is a cellular organelle that can also trigger apoptosis by releasing cell death signaling cascades upon intrinsic or extrinsic stimulus 26. Mitochondrial activities are commonly influenced in neoplastic cells, and targeting this organelle allows for triggering different cell death pathways 11. Furthermore, mitochondria failure may raise the consumption of cell energy supplies and exerts influence in signaling transduction, which lowers the chances of developing drug-resistance 27,28. Considering the potential affinity between peptide-organelle for constructing active targeting drugs, the KLA peptide, an amphiphilic α-helical pro-apoptotic sequence, can interact with the highly negative charged mitochondrial membrane and promote its collapse 29. This cationic peptide sequence is recognized for interacting and disrupting prokaryotic cytoplasmic membranes, which has been reported as a potent weapon in fighting against different cancer cells in breast (MCF-7) 25, brain 28,29, prostate 30, and others commonly affected organs 31. Nonetheless, KLA presents a poor capability of entering eukaryotic cells and thus, depends on a membrane-permeable vehicle for efficient intracellular delivery of the therapeutic 3,5,8,25,28. Strategies such as the use of cell-penetrating peptides (CPPs), including TAT 11,28 and R7 32, nanoparticles 4, homing peptides, and targeting agents, as LTVSPWY 5, HPRP-A 11, folic acid (FA-receptor) 25, and G3-C12 30, showed to overcome the problem of cell internalization of KLA. More recently, cysteine-rich peptides (CRPs) have been identified as potential mitochondrion-targeted molecules 33. However, these strategies cannot always effectively deliver KLA peptides to the targeted mitochondria organelle, where additional conjugation using subcellular targeting molecules or construction peptidomimetic nanoassemblies may be required for a proper therapeutic activity under lower dosages 17,33. The use of higher drug concentrations raises the chances of damaging both normal and cancer cells, which may lead to deeper injures in healthy tissues. Hence, reducing the needed doses by increasing the interaction of active domains with targeted membranes and organelles is a promising strategy for decreasing the collateral effects of chemotherapies 5.

Nanoparticle-mediated drug delivery promises to overcome common constraints correlated to ordinary drug delivery systems, such as limited drug solubility and high dosages 34. They possess large drug-loading capacity due to their multiple attaching sites. Furthermore, nanoparticles can be easily modified with desired cell-targeting moieties 34. An approach that has gained attention in the last years is cancer nanotheranostics, where both diagnostic and therapeutic features are combined, often comprising inorganic and organic portions for developing hybrid nanostructures 35,36. In this context, quantum dots (QD) are among the most applied inorganic nanoparticles for bioimaging, cell targeting, and drug delivery. Organic dyes are still the chief applied entity for bioimaging aims; however, QDs offer advantages such as photostability, broad excitation spectra, sharp emission spectra, and large Stocks shifts 37. The progress in conjugation chemistry enlarged the range of molecules that can be used for modifying QD surfaces, allowing for synergistic functionalities by multivalent targeting 38. QDs can nowadays be conjugated with bioactive molecules, such as drugs, antibodies, and targeting peptides for being tracked for hours-long time intervals, which make possible following all stages of molecule delivery 39. Moreover, QDs have been associated with CMC acting simultaneously as capping ligand and versatile polymer macromolecule for functionalization with biomolecules (e.g., amino acid, peptides) through chemical grafting. This innovative approach produced hybrid colloidal supramolecular nanostructures aiming at cancer nanomedicine and nanotheranostics 22-24. Thus, QDs can be explored as dual-function tools that enable the delivery of the conjugated bioactive moieties while simultaneously provides the visualization of cell-uptake and organelle-delivery of the nanoconjugate 40.

Herein, we report the synthesis of nontoxic AgInS_2_ (AIS) QDs capped with CMC polysaccharide ligand hybridized with L-cysteine amino acids for producing macromolecular structures with enhanced capability for interactions towards Cys-rich domains of cancer cell membranes. The resulting supramolecular nanostructures were conjugated to the pro-apoptotic KLA peptide aiming at an anticancer activity by mitochondria membrane disruption. The newly designed bifunctional QD@polysaccharide-amino acid-peptide nanohybrid, produced *via* green aqueous processes, presented remarkable death-inducing activity towards malignant glioma cells while providing real-time fluorescent bioimaging. Remarkably, the presence of Cys residues tuned the anticancer activity of the nanohybrid in ~70-fold compared to the analogous sample with no Cys residues. Besides, when combined with the drug doxorubicin (DOX), the resulting nanohybrid prodrug presented a synergetic effect as regards cell selectivity towards glioblastoma cells. We anticipate that the results reported throughout this work offer a great foundation for the development of a theranostic nanoplatform, holding great potential for fighting against brain cancer.

## Results and Discussion

### Design of peptidomimetic nanoassemblies

**Figure 1:**
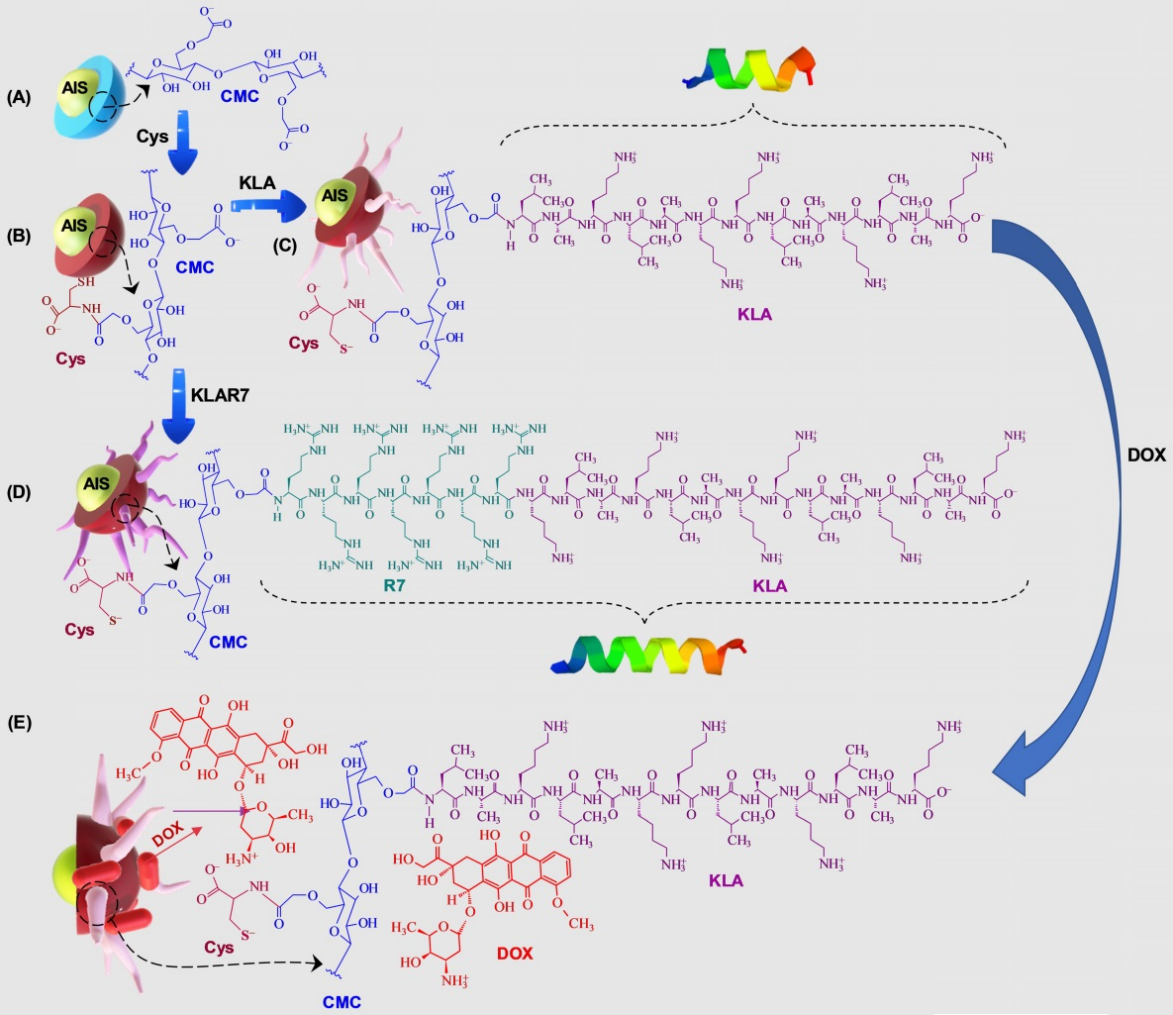
Schematic representation of the steps of construction of the designed nanoassemblies: (A) AIS@CMC, (B) AIS@CMC_Cys, (C) AIS@CMC_Cys_KLA, (D) AIS@CMC_Cys_KLAR7, and (E) AIS@CMC_Cys_KLA-DOX. The helicoidal structures of the peptides were developed by *in silico* simulation.

In this research, peptidomimetic-based nanoassemblies were originally designed to amalgamate five distinct components in an integrated architecture, as schematically depicted in Figure 1.

(i) AgInS_2_ quantum dots (AIS QDs), the inorganic portion of the hybrid nanocarrier, were used as bi-functional nanomaterial biomarkers for tracking nanoconjugate uptake into cells relying on its fluorescence emission.

(ii) carboxymethylcellulose biopolymer (CMC, Figure S1A - Supplementary Material) was used as a water-soluble stabilizing agent of AIS QD (referred to AIS@CMC, Figure 1A) and providing functional groups for covalent bonding with amino acids and peptides mediated by 1-ethyl-3-[3-dimethylaminopropyl] carbodiimide hydrochloride (EDC) chemistry. This type of linkage involving the coupling of the carboxylic/carboxylate groups of CMC with the amine groups from bioactive molecules through an amide bond was chosen as it is one of the most stable linkages in the intracellular environment, also presented in proteins and peptides (peptide bonds) such as the KLA and KLAR7 molecules. Upon internalization of the nanoassemblies through the endocytic pathway, the KLA pro-apoptotic peptide does not need to be released from the nanoconjugates into the endo-lysosome vesicles before delivering inside the cell compartment for causing mitochondrial dysfunction 22.

Moreover, considering the perspective of future clinical applications of these nanosystems, CMC properties were specifically selected to prevent nanoconjugates from rapid blood clearance and elimination from the body (molar mass, M_w_ = 250 kDa) and to favor biodegradability (degree of substitution, DS = 0.7). Theoretically, by improving circulation times, nanocarriers can accumulate in tumors due to the so-called “enhanced permeability and retention” (EPR) effect increasing the therapeutic action *in vivo* 41.

(iii) L-cysteine (Cys, Figure S1D(a)) was selected as a membranotopic vector because its -SH groups can potentially interact with thiol-rich membrane receptors (*e.g.,* integrins), which are overexpressed by cancer cells (*e.g.,* GBM cells) and other exofacial thiol domains and behave as cell-penetrating moieties enhancing nanohybrid internalization. AIS@CMC_Cys (Figure 1B) thiomer derivative was obtained *via* amide bonds (COO^-^ from CMC and NH_3_^+^ from L-cysteine) with a degree of functionalization of 4.2 ± 0.3% estimated by the Ellman's Method.

(iv) KLA peptide (sequence: LAKLAKKLAKLAK, Figure S1B, where L = leucine; A = Alanine, and K = lysine, Figure S1D) was used as a payload to kill cancer cells via mitochondria disruption pathways. As previously described, KLA was covalently bonded to AIS@CMC_Cys (referred to as AIS@CMC_Cys_KLA, Figure 1C) using EDC-chemistry, allowing its release inside the cell cytosol, followed by migration to reach the mitochondria organelle for inducing cell death. Upon conjugation to the CMC polymer, it is also expected to favor the peptide susceptibility to enzymatic degradation 42.

(v) Doxorubicin (DOX) was selected as it is considered the “gold standard” model drug for cancer chemotherapy 22,41 that migrates toward the cell nucleus causing DNA dysfunction by forming a stable drug-DNA complex. In addition, DOX possess intrinsic orange-red self-fluorescence that enables investigation of drug distribution inside cells and tissues through bioimaging techniques 22. In this study, considering that DOX is a cationic drug (pKa ~ 8.3), it was complexed with anionic nanoconjugates predominantly through electrostatic interactions with carboxylate groups (CMC, pKa ~ 4.6), forming hybrid polymer-prodrug nanostructures (AIS@CMC_Cys_KLA-DOX, Figure 1E). These complexed nanocarriers were expected to trigger cancer cell death by comprising the cell-penetrating activity of Cys-bearing thiomer, the KLA peptide for mitochondrial dysfunction, and the DOX chemotherapeutic effect.

As reference samples, two groups of nanoassemblies were also constructed. First, AIS@CMC_KLA samples were produced as “negative reference” lacking L-cysteine as cell-penetrating moiety. For “positive reference”, KLA linked to R7 (KLAR7, sequence RRRRRRRKLAKLAKKLAKLAK, Figure S1C, where R = L-arginine, Figure S1D), which is a globally known CPP, was covalently bonded to AIS@CMC (referred to as AIS@CMC_KLAR7) and AIS@CMC_Cys (referred to as AIS@CMC_Cys_KLAR7, Figure 1D) nanoconjugates. The complete set of samples evaluated in this study is summarized in Table 1.

Prior to the chemical covalent coupling of KLA and KLAR7 to the pristine nanoconjugates (AIS@CMC and AIS@CMC_Cys), their physicochemical parameters, such as isoelectric point and net charge under physiological conditions, were simulated through *in silico* modeling by the *peptide property calculator* (PepCalc.com, Innovagen AB). These results (Table 2) were applied for regulating the functionalization procedures for chemically tethering KLA and KLAR7 to the nanoconjugates and favor the coupling at the N-terminus that is reported to reinforce the stability of the peptides against enzymatic degradation and to augment their metabolic stability 43.

### Characterization of AIS@CMC quantum dots

**Figure 2:**
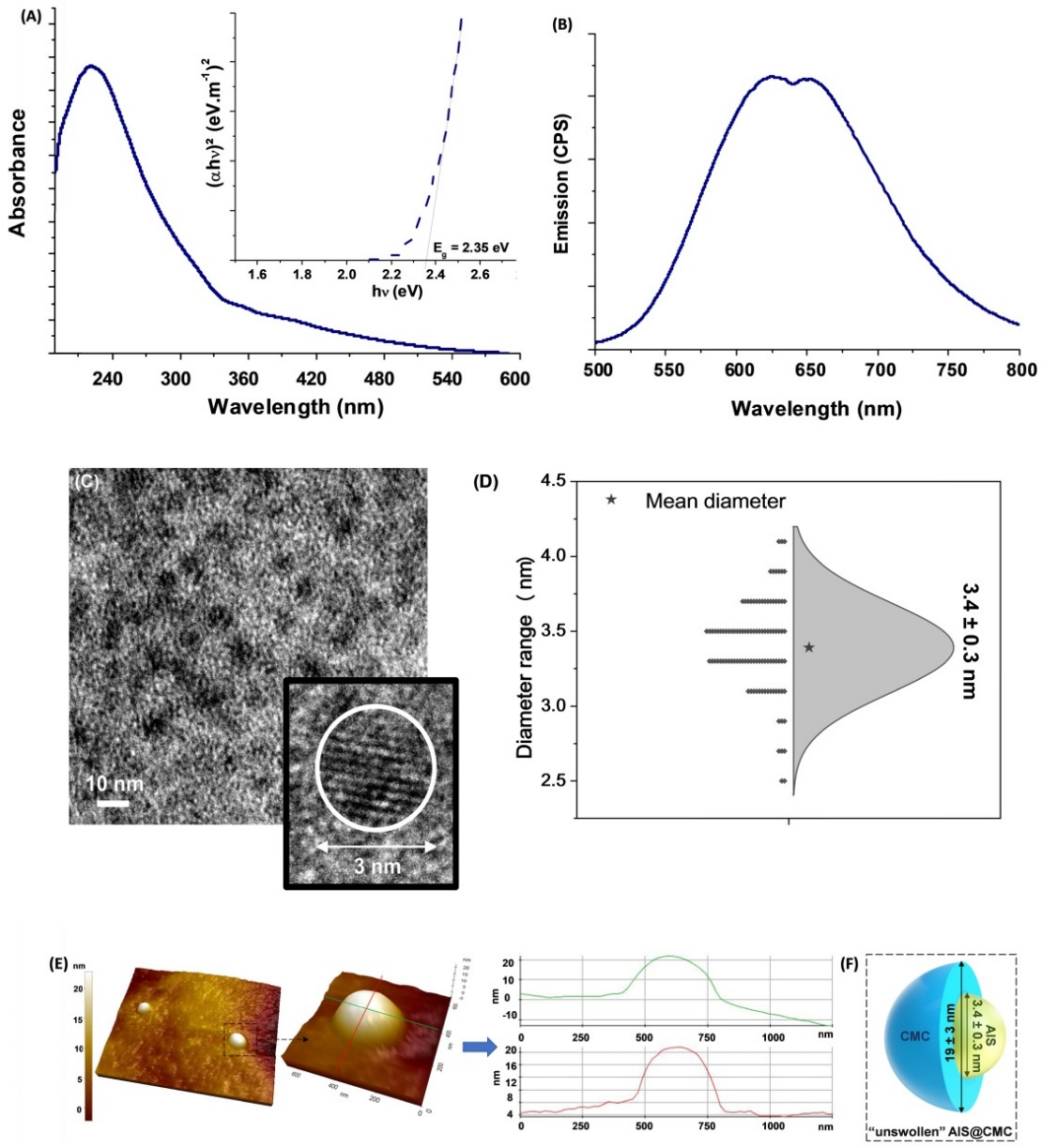
AIS@CMC characterization: (A) UV-vis absorption (inset: Tauc plot) and (B) emission spectra. (C) TEM and HRTEM (inset) images. (D) Histogram of the size distribution of core. (E) Typical 3D topographic AFM image with line profiles. (F) Schematic representation of the core-shell dimensions according to the results of TEM and AFM microscopies (not to scale).

The UV-Vis absorption spectrum of AIS@CMC quantum dots (Figure 2A) presented a broad and featureless curve without a clearly noticeable exciton transition, common to most I-III-VI_2_ ternary semiconductor nanocrystals 44. The bandgap energy (E_g_) was estimated to be ∼ 2.35 eV using “TAUC relation” (inset) for direct bandgap semiconductors, although the E_g_ value cannot be exactly assigned due to the presence of intra-band optical transitions. They mostly arise from the defect states, which result in the tail extending out into the higher wavelengths consistent with chalcopyrite-type nanocrystals. This value (E_g_ ≥ 2.35 eV) was *“blue-shifted”* in comparison to AIS *“bulk”* values, which varies from 1.8 eV to 2.1 eV depending on their crystalline structure 45, thus confirming that all AIS@CMC were in quantum confinement regime 44.

Regarding emission properties (Figure 2B), broad photoluminescence spectra were observed spanning from green to near-infrared (NIR) with two main emission peaks, centered at approximately 625 nm (E_PL_~2.0 eV) and 650 nm (E_PL_~1.9 eV), revealing the existence of at least two major emission pathways. The Stokes shifts (estimated as E_g_-E_PL_) were in the range of 350-450 meV, suggesting that the photoluminescence transitions were based on intrinsic defects (vacancies, interstitials, and antisites) such as donor-acceptor pairs, with the absence of band-to-band excitonic emission 46,47.

The analyses of the morphological features and surface composition of the inorganic QD cores are also important in the development of nanoconjugates for theranostic applications due to their effects on the optical, electronic, and physicochemical properties 37,48. Hence, aiming at assessing size and size distribution of AIS ternary semiconductor core, transmission electron microscopy (TEM) images were obtained (Figure 2C), and revealed the predominance of well-dispersed spherical nanoparticles. In addition, the HRTEM image indicated the crystalline nature of the nanoparticles due to the presence of uniform spaced lattice fringes. The size-distribution diagram (Figure 2D) indicated an average diameter = 3.4 ± 0.3 nm with a relatively monodisperse size distribution (Polydispersity Index, PdI = 0.008), consistent with the criteria in the literature 49. Two different values of the Bohr radius were reported in the literature for AgInS_2_ (2R_Bohr_ = 7.2-7.3 nm 46 and 2R_Bohr_ =11.0 nm 47). Therefore, the TEM findings confirmed that the AIS nanoparticles were produced with an average size much smaller than the Bohr radii, endorsing the quantum confinement regime.

Images of atomic force microscopy (AFM) endorsed the formation of round shaped nanoparticles embedded in a CMC polymer “matrix”, with the typical topographic profiles (Figure 2E). In this sense, from the AFM profiles, the samples presented an average diameter of 19 ± 3 nm obtained from the z-axis values, which endows the best resolution. The relatively larger dimension of AFM measurement compared to the TEM technique was credited to the combination of the polymer shell of CMC (not solvated) surrounding the inorganic AIS core assessed via AFM, which is not commonly observed by TEM due to low contrast limitations (Figure 2F).

In addition to morphological characterization, energy-dispersive X-ray spectra (EDX) and X-ray photoelectron spectroscopy (XPS) (Figure S2) were performed for investigating the chemical composition of the semiconductor core of AIS@CMC nanoconjugates as well as the surface and interface features. As expected, the elemental analysis (Figure S2A) revealed the presence of Ag, In, and S in the ternary Ag-In-S QD nanoalloys. The estimated molar ratio of metallic precursors was [Ag]:[In] = 1:4.3 and [Ag]:[In] = 1:4.5, by EDX and XPS, respectively, which are consistent with the molar ratio of precursors used during the synthesis of [Ag]:[In] = 1:4. This chemical composition (off-stoichiometry) was chosen because it gives the most intense emission for AgInS_2_ nanocrystals, assigned to the population of intra-band defect states that predominantly governed AIS photoluminescent behavior 50. XPS analysis was also performed to assess the surface chemistry of nanoconjugates as well as to investigate the oxidation states of the chemical elements of the inorganic core. The XPS spectrum of Ag 3d region (Figure S2B) revealed a doublet at 374.1 eV (Ag 3d_3/2_) and 368.2 eV (Ag 3d_5/2_) that was associated with Ag (3d) transitions in Ag^+^, with a spin-orbit splitting of 5.9 eV. For In 3d region (Figure S2C), the peaks at 452.2 eV and 444.5 eV correspond to the 3d_3/2_ and In 3d_5/2_ levels, respectively, typical of In^3+^ transitions. A binding energy interval of 7.7 eV separated the spin-orbit components. Moreover, S 2p (Figure S2D) signals showed two overlapped peaks at 162.6 eV (S 2p_1/2_) and 161.4 eV (S 2p_3/2_), with Δ = 1.2 eV, which are characteristic of sulfides (S^2-^) 45,51.

These findings demonstrated that ultra-small ternary AIS quantum dots were formed and stabilized by CMC macromolecular ligand as colloidal nanoconjugates (AIS@CMC), uniformly monodispersed in an aqueous medium with morphological features potentially feasible for applications in nanomedicine 23,44,50.

### Characterization of peptidomimetic nanoassemblies

#### Fourier transformed infrared spectroscopy (FTIR) and proton nuclear magnetic resonance of bioconjugates (H-NMR)

**Figure 3:**
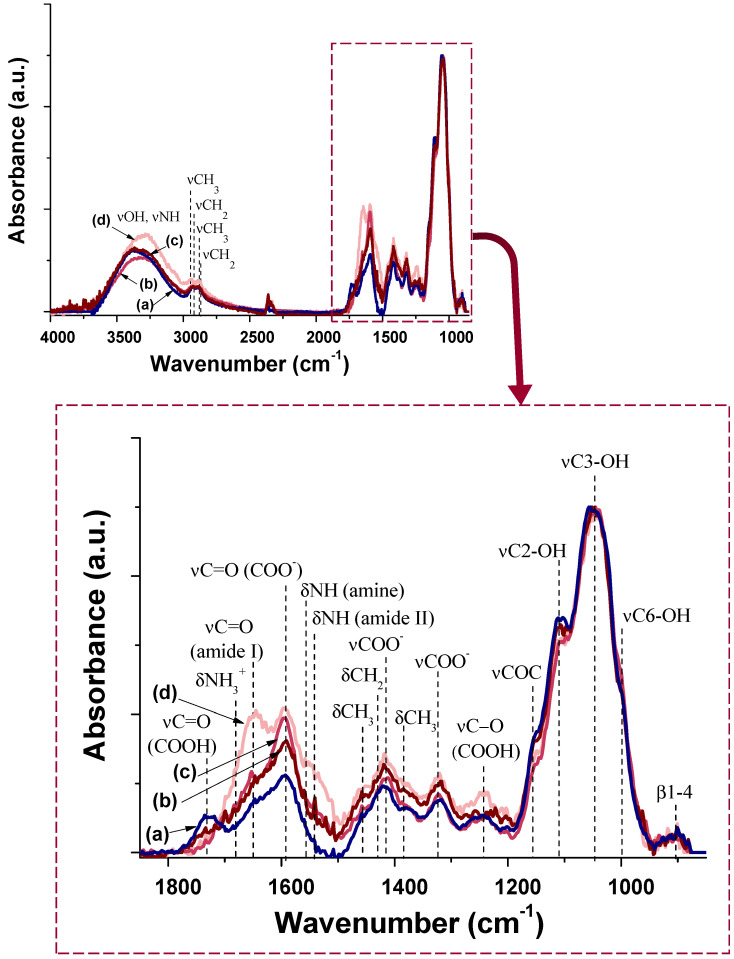
FTIR spectra of nanoconjugates: (a) AIS@CMC, (b) AIS@CMC_Cys, (c) AIS@CMC_Cys _KLA, and (d) AIS@CMC_Cys_KLAR7.

The structural characterization by Fourier transformed infrared spectroscopy (FTIR) was performed to evaluate the changes in the AIS@CMC nanoconjugates upon hybridization with cysteine (Cys) amino acid and tethered with KLA and KLAR7 peptides. In all of the spectra (Figure 3 and Figure S3), the typical bands of CMC capping ligand associated with hydroxyls (-OH), methylene (-CH_2_), carboxylate/carboxylic (-COO^-^ /-COOH), alcohols (C-OH), and β1-4 glycoside bonds were observed. The grafting of AIS@CMC (Figure 3a) with Cys was mainly identified by the presence of the amide I band at ca. 1650 cm^-1^ in the spectrum of AIS@CMC_Cys (Figure 3b), which was formed between carboxylate groups of CMC and amine groups of Cys during the functionalization based on the EDC-chemistry process. The vibrational band associated with the thiol side groups (*ca.* 2550 cm^-1^) of Cys is often very weak, and therefore, it is scarcely observed by FTIR. The covalent bonding of KLA and KLAR7 peptides to AIS@CMC_Cys (Figure 3c and Figure 3d, respectively) and AIS@CMC (Figure S3b and Figure S3c, respectively) was verified by the increase/appearance of amide I (~1650 cm^-1^) and amide II (~1540 cm^-1^) bands, that are related to both the coupling reaction between carboxylate groups of AIS@CMC_Cys (or AIS@CMC) and the N-terminal amine groups of the peptides and to the peptide bonds in KLA and KLAR7 sequences.

Also, the presence of remaining amine (-NH_2_) groups in lysine residues after conjugation was identified by the significant increase of absorbance at 3000-3300 cm^-1^, ascribed to νNH, and at 1555 cm^-1^, which is related to δNH vibrations. Moreover, the increase in the absorbance values at 2900-3000 cm^-1^, at 1450-1470 cm^-1^, and at 1380 cm^-1^ were assigned to νCH_3,_ asymmetric δCH_3_, and symmetric δCH_3,_ respectively, present in both alanine and leucine residues 52. Regarding KLAR7-modified nanoconjugates, the changes in the amide bands were more prominent than unmodified analogs due to the contributions of seven arginine residues (R7) in each molecule, which raised the number of peptide bonds per ligand chain. Additionally, signals relative to NH_3_^+^ asymmetric deformation of guanidyl groups were identified at 1680 cm^-1^ 23,52.

**Figure 4:**
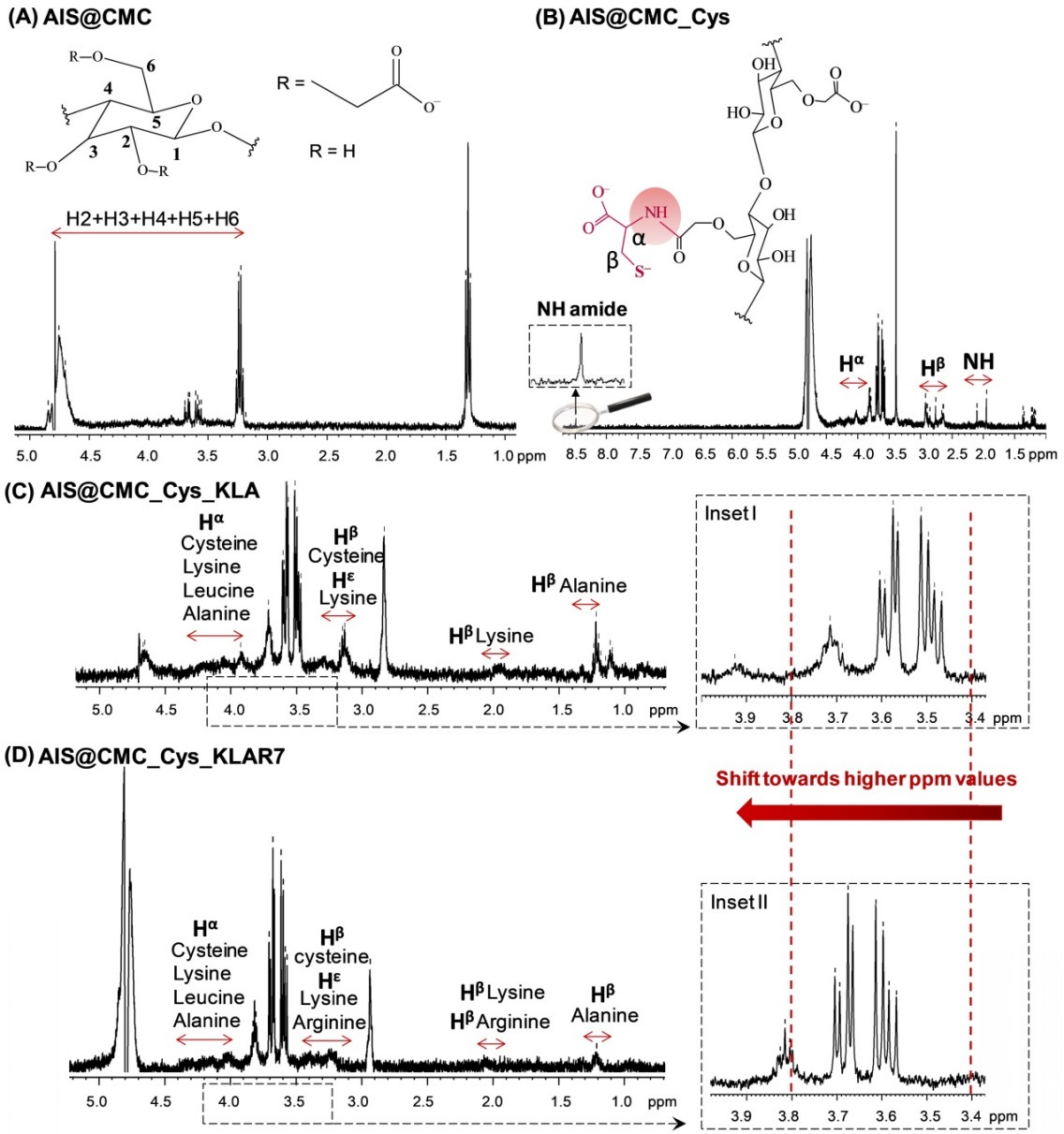
^1^H-NMR spectra of (A) AIS@CMC, (B) AIS@CMC_Cys (C) AIS@CMC_Cys_KLA, and (D) AIS@CMC_Cys_KLAR7.

For further demonstrating the functionalization of the CMC backbone with Cys amino acid and/or KLA and KLAR7 peptides, the proton nuclear resonance spectroscopy (^1^H-NMR) spectra of all samples were also collected (Figure 4 and Figure S4). The ^1^H-NMR spectra of AIS@CMC, AIS@CMC_Cys, AIS@CMC_Cys_KLA, and AIS@CMC_Cys_KLAR7 are shown in Figure 4A-D, respectively. Protons related to the CMC backbone were identified in the ^1^H-NMR spectra of all AIS@CMC-based nanoconjugates, where the signals from 3.5 - 5.0 ppm are associated with C-H protons in C2-C6, and variations in those peaks are credited to the chemical substitution in the cellulose backbone 53.

The formation of amide bonds between CMC ligand and Cys was identified through the peak at 8.5 ppm in Figure 4B, which is related to the NH proton of the amide bond. Moreover, peaks at ~2.0 ppm, 2.7-2.9 ppm, and 3.9-4.2 ppm were respectively associated with nitrogen-linked hydrogen, to methylene protons of β-carbon, and α-Carbon. The appearance of peaks associated with protons of cysteine, alanine, lysine, and leucine, which are highlighted in the spectra of Figure 4C-D, demonstrated the functionalization of AIS@CMC_Cys with KLA and KLAR7 peptides 54. For all samples based on AIS@CMC functionalized nanoconjugates, chemical shifts towards higher ppm values were detected in the region of H2-H6. These shifts can be related to the formation of new hydrogen bonds between the functionalized CMC chains and to the modification of the chemical environment of protons due to the insertion of new functional groups onto CMC. The proximity between oppositely charged chemical groups (negative CMC chains and positive KLA or KLAR7 peptides) allows for charge transfer and for strengthening the dispersion forces. Consequently, there is a decrease in the electron density around the H nucleus, and the chemical signals shift toward higher ppm values (see insets I-II in Figure 4C-D). It is necessary to mention that the chemical shifts of amino acid protons can vary depending on the local structure and neighboring amino acid residues 54. Similar behavior was detected in the ^1^H-NMR spectra of AIS@CMC_KLA and AIS@CMC_KLAR7 presented in Figure S4. The chemical structures of individual amino acids that constitute the apoptotic peptides, as well as of KLA/KLAR7 sequences, were presented in Figure S1.

Hence, the results of FTIR and ^1^H-NMR spectroscopic analyses confirmed the effective functionalization of the pristine AIS@CMC with Cys and/or KLA or KLAR7 molecules through the formation of amide bonds.

#### Physicochemical characterization by dynamic light scattering and zeta potential analyses of bioconjugates

Aiming at evaluating the influence of nanoconjugate functionalization on hydrodynamic diameter (D_H_) and overall surface charge, dynamic light scattering (DLS) and zeta potential (ζ, ZP) analyses of samples were performed. Both KLA and KLAR7 peptides are positively charged and considerably large molecules, possessing approximately 1.5 kDa and 2.5 kDa, respectively (Table 2). Thus, the functionalization of AIS@CMC (D_H_ = 74 nm, PdI = 0.385; ζ = - 49 ± 3 mV) and AIS@CMC_Cys (D_H_ = 66 nm, PdI = 0.247; ζ = - 18 ± 1 mV) with these molecules is expected to cause a substantial effect on the physicochemical properties of the nanoassemblies. As expected, upon conjugation with the peptides, an increase of D_H_ was observed in comparison to pristine nanoconjugates. Although comprising a peptide with a much higher molar mass, KLAR7-functionalized nanoconjugates showed to be more compact nanocolloids (AIS@CMC_KLAR7: D_H_ = 95 nm, PdI = 0.330; AIS@CMC_Cys_KLAR7: D_H_ = 99 nm, PdI = 0.370) compared to those functionalized with KLA peptide (AIS@CMC_KLA: D_H_ = 141 nm, PdI = 0.336; AIS@CMC_Cys_KLA: D_H_ = 139 nm, PdI = 0.311). This trend was attributed to the grafting of positive arginine-sequences in KLAR7 (net charge 13) as the CPP agent, which may have interacted with the negatively charged carboxylate groups of AIS@CMC and AIS@CMC_Cys nanoconjugates, leading to conformational changes and reduction of the hydrodynamic size.

Regarding surface charge balance, it is noteworthy the effect of hybridization with Cys in reducing the zeta potential of AIS@CMC conjugate from - 49 mV to -18 mV. This effect on the ZP was ascribed to the presence of Cys residues in AIS@CMC_Cys, causing depletion in the number of charged chemical groups on the surface of the nanoconjugate 23. Therefore, in the case of AIS@CMC, modification with KLA (ζ = - 41 ± 5 mV) or KLAR7 (ζ = - 10 ± 1 mV) caused more prominent changes in zeta potential compared to the functionalization of AIS@CMC_Cys with the same molecules (KLA-modified, ζ = - 13 ± 1 mV; KLAR7-modified, ζ = - 23 ± 5 mV). Conversely, the interactions between AIS@CMC_Cys and the inserted peptides are not as intense as in the case of the highly negative AIS@CMC. As AIS@CMC_Cys does not possess a strong negative surface charge, the electrostatic interactions between AIS@CMC_Cys and KLA were not extensive and, therefore, did not cause an important variation in ζ values. This conservation in the ζ-values, even with the insertion of highly positive moieties, is supporting evidence of the stabilization of the thiolate groups in Cys residues due to the presence of electron positive neighbor groups and hydrogen bonding, which lowers the pKa of thiol 13. It should be noted that, even though AIS@CMC_KLA and AIS@CMC_Cys_KLA nanoconjugates possess similar values of D_H_, their surface charges (ζ) are substantially different. This feature raises an important effect regarding the spatial configuration of the KLA peptide when grafted to the CMC-based macromolecule. While the *ζ*-value of AIS@CMC_KLA may be predominantly governed by electrostatic repulsion between negatively charged COO^-^ groups, the *ζ*-value for AIS@CMC_Cys_KLA nanoconjugates would be associated with a configuration balanced by attractive interactions between the inserted thiol groups from cysteine with carboxylates from CMC. Table 3 summarizes the results of DLS and ZP analysis and, according to the literature, the values of D_H_ (< 200 nm) and PdI (< 0.385) obtained for the nanoconjugates are appropriate for intravenous administration and for brain tumor targeting by the EPR effect 55.

#### Optical properties of AIS-CMC-Peptide nanoassemblies

The photostability and brightness of QDs associated with their capability of being modified with specific ligands, like proteins, bioactive moieties, and peptides, make them remarkable nanomaterials for producing fluorescent labels for bioimaging and cell targeting 39. Moreover, structural modifications in capping ligands are known to largely influence the optical properties of QD-based nanostructures, which can be suitable as nanoprobes for investigating cellular events and tracking signaling pathways 48,56. However, the prediction of the effects caused by ligand functionalization is still a great challenge because of the complexity of the phenomena involved. Emission properties of QD-based nanoparticles, such as fluorescence lifetimes, are often affected by bioconjugation. Conversely, absorption and emission spectral shapes are less influenced by surface modification 57. Thus, we endeavored to assess some of the most relevant optical features of AIS-CMC nanoconjugates associated with their peptide modifications.

**Figure 5:**
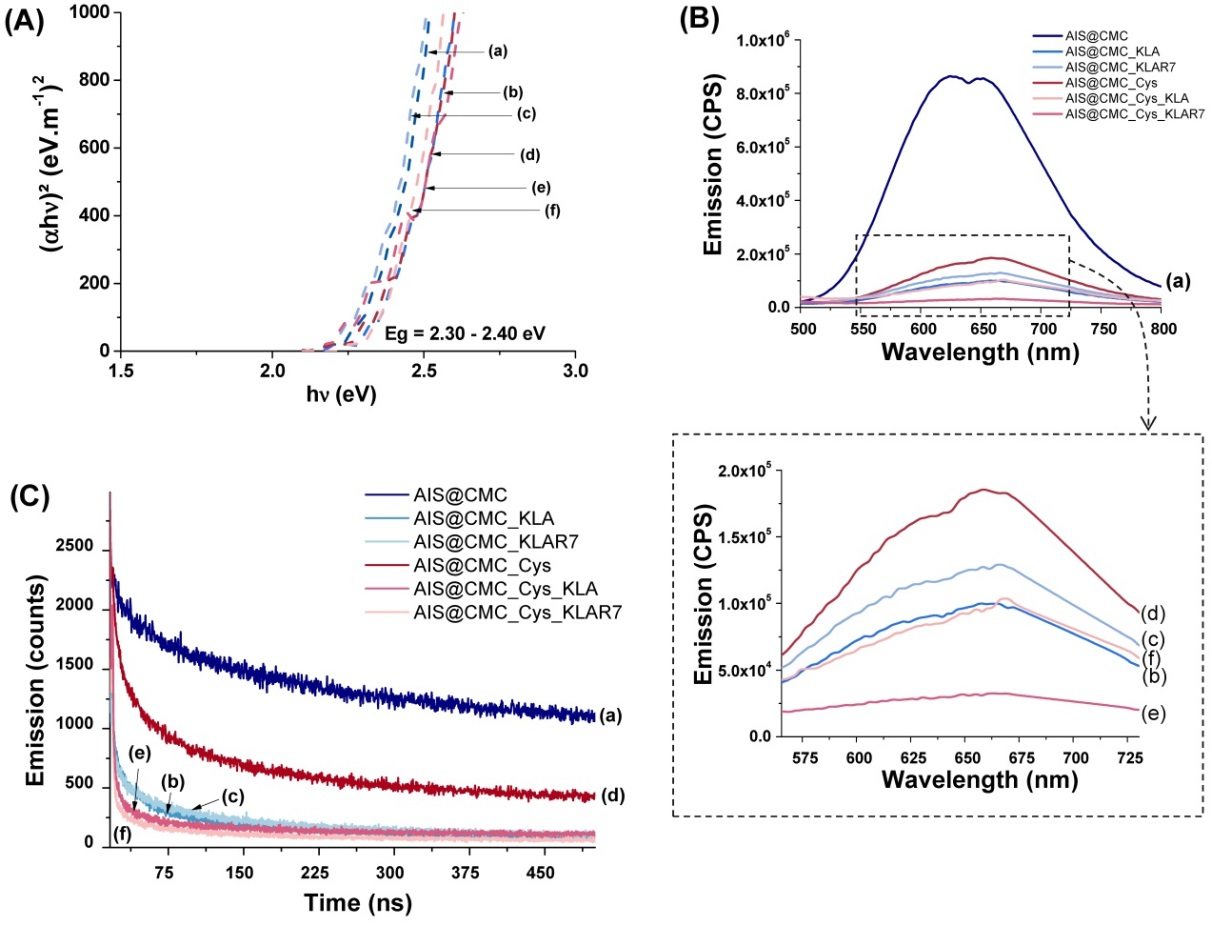
(A) Tauc curves, (B) Emission spectra (λ_excitation_ = 350 nm), and (C) Emission decay curves (λ_excitation_ = 375 nm and λ_emission_ = 625 nm) for (a) AIS@CMC, (b) AIS@CMC_KLA, (c) AIS@CMC_KLAR7, (d) AIS@CMC_Cys, (e) AIS@CMC_Cys_KLA, and (f) AIS@CMC_Cys_KLAR7.

Based on UV-vis data, no significant differences in the bandgap values (E_g_) were detected for the chemically functionalized nanoconjugates. Tauc curves (Figure 5A) indicated that the E_g_ values ranged from 2.30 to 2.40 eV, similar to non-modified AIS@CMC, in agreement with the literature 58.

The emission spectra of all samples (Figure 5B) showed similarities regarding shape and spectral position within the wavelength ranging approximately from λ=500 to λ=800 nm related to the photoluminescence. However, a considerable quenching of QD core emission intensity was noticed for amino acid and peptide-functionalized nanoconjugates (Figure 5B(b-f)) compared to the unmodified AIS@CMC (Figure 5B(a)). Change in the PL intensity is a common effect observed for chemical modifications of capping ligands and is often associated with energy transfer between the fluorescent semiconductor inorganic cores and surrounding organic groups, which can be explored as optical nanosensors 23,24,48. Thus, these results have given supporting evidence that the functionalization of biological molecules to AIS@CMC conjugates have occurred through the significant changes detected in the optical properties of the nanoassemblies.

The quantum yield (QY) of approximately 3% was measured for AIS@CMC nanoconjugates, which is well-matched with ternary Ag-In-S QDs synthesized via the aqueous colloidal process. As observed in the PL spectra previously discussed, the functionalization of AIS@CMC nanoconjugates caused important quenching of the emission intensities of the nanoassemblies, consequently leading to the reduction of QY (AIS@CMC_Cys_KLA, highest reduction of ∆~90%). Nonetheless, it is well-established in the literature that, due to the high extinction coefficient, water-soluble quantum dots with low QY (~ 0.2%) have been successfully applied for numerous applications in bioimaging and nanosensors 59.

PL emission decay is another key optical feature for QDs applied for bioimaging. Thus, time-correlated single-photon counting (TCSPC) measurements were performed for all samples at λ_emission_ = 625 nm (Figure 5C). The decay curves of AIS@CMC and AIS@CMC_Cys typically followed a three-exponential model, in accordance with long-life QD fluorophores, which normally present multi-exponential decays 57. For bioconjugates, decay curves were fitted by four-component exponentials. This characteristic indicates the presence of an extra relaxation pathway for the peptide-modified nanoconjugates, which was ascribed to the additional electronic transitions from electron-rich groups (heteroatoms, N, S, and O) grafted to the macromolecular structure favoring donor-receptor exchange. This phenomenon is in agreement with new bands/peaks observed in the UV-vis and PL emission spectra. The values of intensity averaged lifetimes (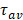) were summarized in Table 4, and the decay times (τ_i_) and amplitude ratios (A_i_) of the PL emissions were presented in Figure S5. As a general trend, the reduction of the PL-lifetime was observed for all modified samples in comparison with unmodified AIS@CMC conjugates. These results corroborate the hypothesis that the changes detected in emission intensities are predominantly related to energy transfer between the AIS cores and the modified ligands 24,57. Moreover, all lifetimes are considerably longer than those of commonly reported organic fluorophores, which are typically bellow 10 ns 57. To this end, theoretically, the longer lifetimes verified for these nanoassemblies allow for better temporal separation of the QD signals from cellular auto-fluorescence, boosting bioimaging sensitivity 57, as well as permitting long-term tracking in biological studies 60.

### Biological behavior of peptidomimetic nanoassemblies

**Evaluation of cytotoxicity of nanoconjugates by mitochondrial activity cell viability assay (MTT)**. *Rationale*: It is important to call attention to the fact that there are two major pathways leading to caspase activation and cell death by apoptosis. One comprises the death receptor pathway (e.g., tumor necrosis factor receptor), and the second involves the mitochondrial pathway (e.g., pro-apoptotic molecules) 61. Triphenylphosphonium-based compounds (TPPs) have been widely used as a mitochondria-targeting vector 33. Essentially, the hydrophobicity associated with this lipophilic cation favors its interaction with the hydrophobic inner mitochondrial membrane and facilitates its penetration into the mitochondria. However, the conjugation process most frequently is based on organic routes mediated by crosslinkers through more intricate coupling protocols. Additionally, it has been reported that the mitochondrial accumulation of TPP can adversely affect mitochondrial function and induces cytotoxicity, limiting their potential therapeutic applications. 33. On the other hand, peptide-based targeting vectors, including cell membranes and organelles, can be readily tuned for specific applications by incorporating various monomers and sequences at the preparation stage that employs a relatively simple synthesis technique. To this end, KLA peptide is known as a very effective pro-apoptotic molecule to trigger cell death through the disruption of mitochondrial membranes. However, it lacks the ability to permeate eukaryotic cell membranes and present insufficient localization in mitochondria, demanding the aid of coupled cell-penetrating molecules or tracking species to properly activate the cell death pathways 11,30. For that reason, cell-penetrating peptides (CPPs) such as R7 (arginine-rich) peptides have been reported to ameliorate both cell uptake and mitochondria targeting, therefore enhancing the therapeutic effects of KLA-bearing drugs 30. Moreover, new studies have emerged investigating the intriguing role of thiol-modified macromolecules with the cysteine-rich domains of cell membrane proteoglycans in facilitating the cell uptake and intracellular traffic of nanostructures, which can represent a prospective targeting strategy in the design of delivery agents for cancer theranostics 62.

Herein, we developed novel hybrid conjugates for studying their capability of acting as intracellular and subcellular delivering agents of KLA-modified nanoassemblies for killing glioblastoma (U-87 MG) cancer cells. It was hypothesized that the functionalization with the CPP sequence (R7) and Cys residues in the CMC polysaccharide ligand would significantly affect the anticancer activity of the hybridized QD-polysaccharide-peptide nanostructures through the enhancement of the cellular uptake. To that end, the most relevant aspects, such as KLA-induced cytotoxicity and relative cell selectivity towards cancerous cells, were comprehensively examined regarding these bioconjugate nanostructures. The cytotoxicity of bare nanoconjugates (AIS@CMC and AIS@CMC_Cys), biofunctionalized with KLA peptide (AIS@CMC_KLA and AIS@CMC_Cys_KLA), and coupled to KLA peptide bound to R7 sequence (CPP, AIS@CMC_KLAR7, and AIS@CMC_Cys_KLAR7) were accessed using 3-(4,5-dimethylthiazol-2-yl)-2,5-diphenyltetrazolium bromide (MTT) bioassays with two different times of incubation (6 h and 24 h), and two concentrations of peptides (0.8 µM and 4 µM). This bioassay was selected not only because it is the most widely accepted test for preliminary assessment of cytotoxicity of nanomaterials and medical devices 63 but also because it relies on the mitochondrial activity that will be affected by our cell death payload (KLA). Furthermore, both HEK 293T (healthy) and U-87 MG (cancerous) cell lines were used in the biological experiments to evaluate potential *in vitro* selectivity of the bioconjugates towards brain cancer cells.

**Figure 6:**
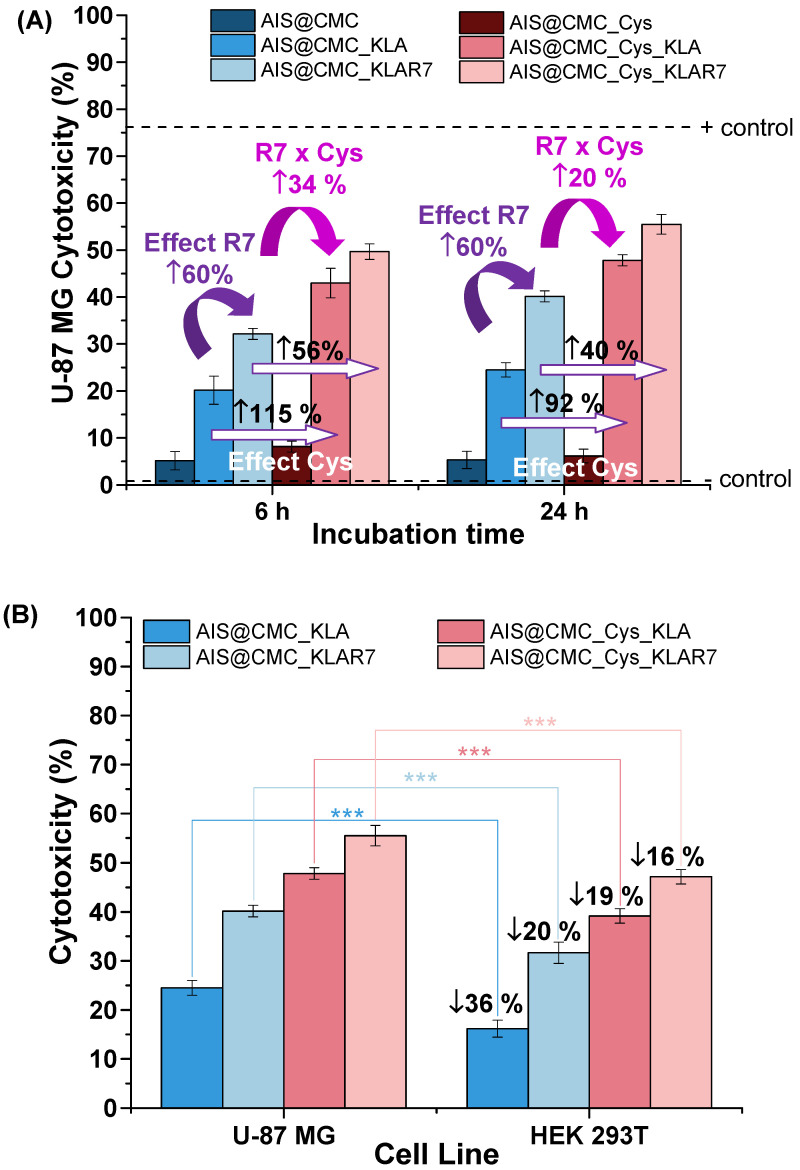
(A) Cytotoxicity responses of nanoconjugates after 6 h and 24 h of incubation with U-87 MG cell line and [peptide] = 0.8 µM. (B) Comparison of toxicity of U-87 MG and HEK 293T after 24 h of incubation with bioconjugates (mean ± standard deviation (SD); n = 6; One-way ANOVA followed by Bonferroni's test with *** = p ≤ 0.001).

The results of cytotoxicity are summarized in Figure 6 (U-87 MG) and Figure S6A (HEK 293T), and additional controls are presented in Figure S6B. As expected, it was observed that the AIS@CMC and AIS@CMC_Cys nanoconjugates were non-toxic to cancerous (< 10%, Figure 6A) and normal (< 10%, Figure S6A) cells, which was attributed to the intrinsic biocompatibility of the CMC polymer ligand as well as the minor toxicity of AIS QDs (core). Moreover, no cell death-inducing effects were observed after grafting Cys residues to AIS@CMC forming thiomeric nanoconjugates, despite the thiol biochemistry in cells being very complex, comprising a fragile counterbalance between their beneficial and pathogenic reactions 13,64. Conversely, as a general trend, the hybridization of AIS@CMC and AIS@CMC_Cys with KLA or KLAR7 peptides triggered a significant cytotoxic effect in both cell lines.

*Effect of KLA and KLAR7*. The presence of KLA peptides grafted to CMC in the nanoconjugates (AIS@CMC_KLA) resulted in cytotoxicity responses below 25% after 6 h and 24 h of incubation with U-87 MG cells (Figure 6A). According to the ISO 10993-5:2009/(R)2014 (Biological evaluation of medical devices: Tests for *in vitro* cytotoxicity), these results indicated that AIS@CMC_KLA is evaluated as non-toxic (*i.e.,* toxicity < 30%). However, these findings revealed the cellular uptake of AIS@CMC_KLA nanoconjugates as the therapeutic action of the KLA peptide depended on internalization promoted by the accessory nanocarrier 65. Thus, although KLA peptide possesses poor cell membrane permeability 3,5,8,25,28, this behavior was credited to the capability of CMC polymer macromolecule forming the nanostructures (AIS@CMC_KLA) to be internalized by cells, supported by previous studies of our group 1,22,50. The additional functionalization of bioconjugates with R7 moieties fused to the macromolecular structure resulted in an increase of cytotoxicity to tumor cells of 6 % for AIS@CMC_KLAR7 compared to AIS@CMC_KLA (toxicity from 20% to 32% and from 25% to 40% after 6 h and 24 h of incubation, respectively), unveiling the remarkable effect of the arginine-rich CPP in the delivery of the nanoconjugates to cancerous cells. According to the literature 29, this effect was attributed to the better interaction of R7-containing samples with overexpressed anionic membrane proteoglycans, such as heparan and chondroitin sulfates 66,67. Also, enhancement of internalization is associated with interactions (hydrogen bonds and ions pairs) of the guanidine groups of arginine with phosphate moieties on the cell membrane 68. These results also demonstrated that both KLA and KLAR7 peptides grafted to the CMC backbone retained the appropriate bioactive conformation and biological activities after the conjugation procedures, which endowed the peptidomimetic behavior of these nanoassemblies.

*Effect of Cys hybridization*. Regarding the Cys-bearing bioconjugates associated with KLA pro-apoptotic peptide (*i.e.,* AIS@CMC_Cys_KLA and AIS@CMC_Cys_KLAR7), the incorporation of thiol groups in the macromolecular structure of the ligands showed a boosting effect on the cytotoxicity (Figure 6A). The lethality induced by AIS@CMC_Cys_KLA was 43% and 48% after 6 h and 24 h of contact with tumor cells, respectively, in comparison to 20% (6 h) and 25% (24 h) observed for the AIS@CMC_KLA. That means the thiolation of CMC provoked a relative increase of toxicity of the KLA-modified conjugates higher than 90% after the incubation with U-87 MG cells. For Cys_KLAR7 nanoassemblies, the cytotoxicity responses of 50% (6 h) and 56% (24 h) were measured with U-87 MG cells, indicating an increase of cell death higher than 40% (∆%) in comparison to AIS@CMC_KLAR7. It is noteworthy that the highest lethality observed for the AIS@CMC_Cys_KLAR7 system was ascribed to the sum of the contributions of KLA as a pro-apoptotic peptide, with Cys and R7 as cell-penetrating agents (Table 1). Moreover, these results demonstrated that the biological function designed for L-cysteine (*i.e.,* thiol-bearing biomolecule) in the bioconjugates as cell-penetrating moiety for augmenting KLA internalization was achieved. This effect was clearly evidenced by the MTT results at 24 h of AIS@CMC_Cys_KLA (cytotoxicity of 48%) in contrast to AIS@CMC_KLAR7 (cytotoxicity of 40%), indicating an increase of cell death of approximately 20% associated with the presence of Cys residues replacing R7, the globally accepted cell-penetrating peptide.

As previously discussed, it is reported in the literature that natural cell-surface thiol-bearing molecules actively intermediate the internalization processes through the dynamics of the redox species (SH/S-S), which have motivated this study of thiol-based cell-penetrating agents as innovative cargo delivery agents 16-18,69. Although the accurate mechanism is yet unknown, the most accepted theories comprise the formation of mixed disulfide complexes between thiols from the cell membrane and delivery agents (balance of reduced/oxidized species) at the biointerfaces, followed by internalization of the complex, and finally, the release of the entities within the reducing environment of cell cytoplasm 16-18,69. The boosting effect of single amino acid residues in the treatment of cancerous cells is of great interest for the progress of effective chemotherapies, although it remains unsolved regarding the contributions of cysteine residues in directing cell death, besides acting as a cell-penetrating agent. Regarding the drug targeting perspective, the mitochondria are known as regulatory centers of cell death and apoptosis and, once destabilized, they release Cytochrome C, inducing the formation of reactive oxygen species (ROS), and the increase in Ca^2+^ levels in the cytosol. Thus, this apoptotic cascade might have influenced the results observed on the boosting effect of Cys residues in cell death induced by KLA. Under oxidative conditions, Cys residues can generate toxic products (*i.e.,* s-sulfocysteine and cysteate), which are critical signaling-intermediates and can affect ionotropic and metabotropic glutamate receptors, mediating the neurotoxic effects in the presence of Ca^2+^ 64. In this sense, in order to evaluate the plausible contribution of the Cys residues in cell death triggered by KLA/KLAR7, further examinations were conducted by the analysis of ROS formation.

Thus, aiming at accessing the accumulation of intracellular ROS inside U-87 MG cells after the nanoconjugate internalization, the measurement of reactive species was performed by staining hydrogen peroxide (H_2_O_2_), hydroxyl (OH^-^), and peroxyl (ROO^-^) radicals with 5-(and -6)-carboxy-2′,7′-dichlorodihydrofluorescein diacetate (DCF-DA) and the results are shown in Figure S7. As a general trend, only a minor increase in intracellular ROS formation was observed compared to the negative control, and the values for the nanoconjugates were more than 20-fold smaller than the positive control (tert-Butyl hydroperoxide, TBHP) after 60 min of incubation of U-87 MG cells with samples. The AIS@CMC and AIS@CMC_Cys nanoconjugates, which demonstrated to be non-toxic according to the above-discussed MTT results, presented similar results regarding ROS formation compared to their KLA and KLAR7 derivatives. Additionally, no significant differences were detected among AIS@CMC/AIS@CMC_KLA/AIS@CMC_KLAR7 and the Cys containing nanoconjugates. Thus, based on these findings of ROS analyses, it is suggested that the oxidative stress pathway was not primarily accountable for the higher lethality observed for these systems *via* MTT assay, neither cell death by KLA or KLAR7. Nonetheless, a more in-depth analysis of other possible complex mechanisms of mitochondria-mediated cellular toxicity promoted by Cys residues conjugated with KLA peptides is out of the scope of this research and, therefore, should be addressed in future studies.

*Effect of KLA concentration*. To validate that the triggering of death induction governed by the KLA peptide was concentration-dependent, samples possessing a 5-fold higher KLA content ([KLA] = 4 µM in comparison to previous results at [KLA] = 0.8 µM) bioconjugated with the same AIS@CMC or AIS@CMC_Cys concentrations were prepared and evaluated by MTT assay. The results (Figure S8) corroborated the death-inducing effect of KLA, the action of Cys residues as cell-penetrating moiety, and an enhanced lethality with increasing the KLA peptide concentration grafted to the nanoconjugates. After 24 h of incubation with U-87 MG cells, cytotoxicity responses reached 35% and 70% for AIS@CMC_KLA and AIS@CMC_Cys_KLA nanosystems with [KLA] = 4 µM, respectively. These results indicated a boost of lethality higher than 40% by increasing the relative concentration of KLA forming the bioconjugates. This outcome revealed the broad range of loading capability as pro-drug delivery agents as well as the possibility of tuning the dosage of the therapeutic by adjusting the grafting extension of peptides while maintaining a constant nanocarrier concentration.

*Effects on Cell Type*. Regarding the cytotoxicity responses of the nanoconjugates towards HEK 293T (normal) cells (Figure S6A), analogous behaviors of peptides and penetrating moieties were verified for this cell line in comparison to U-87 MG cancer cells after 6 h and 24 h of incubation. However, highly important was the fact that the relative lethality of the nanoconjugates was less prominent with the healthy cells compared to the glioma tumor cells, as depicted in Figure 6B. When comparing the cell death induced by the same bioconjugate nanosystem, the differences in cytotoxicity were statistically significant (with p < 0.001), indicating that bioconjugates presented a relative cell selectivity towards cancerous cells. Assuming the ratio of cytotoxicity of cancer cells to normal cells as a preliminary evaluation of “selective index”, the obtained values were 1.51 and 1.22 for AIS@CMC_KLA and AIS@CMC_Cys_KLA, respectively. Based on these findings, even though not definitive, a relative “protective effect” was observed for these nanoconjugates towards normal cells. This is a highly desirable feature for minimizing the side-effects in prospective clinical applications in cancer therapy. This cell-dependent behavior can be interpreted as the contribution of several aspects different from healthy and tumor cells supported by the literature 70-72. From a general perspective, this difference in activity is expected due to variation in cell physiology, metabolism, membrane characteristics of cells and organelles (permeability, stability, presence of receptors, etc.), which also influences endocytosis and exocytosis pathways and kinetics. In this study, an important aspect to be considered is associated with the internalization pathways, as the novel nanoconjugates were constructed using distinct moieties for affecting both the cellular uptake and the intracellular organelle targeting. The endocytotic route is acknowledged as greatly dependent on both the nature of the delivery agent and the composition of the cell membrane, which means that it is highly influenced by the cell line 16-18,69-72. Hence, in the case of glioblastoma cells that overexpress thiol-rich membrane receptors (*e.g.,* integrins), the enhanced uptake observed for the Cys-bearing nanohybrids can be ascribed to the thiol/disulfide exchange reactions (*i.e.,* redox balance) and interactions occurring between sulfhydryl-rich domains at the cell membranes with the chemically-modified bioconjugates. It has been reported that extracellular cysteine-rich domains are potential sites of thiol-disulfide exchanges, although the precise mechanism remains unsolved 73,74. Moreover, another key aspect that cannot be neglected is the higher metabolism of cancer cells in comparison to normal cells. This may have boosted the killing activity of the developed nanoconjugates modified with pro-apoptotic peptides (CMC_Cys_KLA) towards glioblastoma cells 75.

Considering these rationally designed nanoconjugates, the key results presented in this section can be summarized as follows: (i) KLA peptide was used as payload to kill cancer cells; (ii) Cys acted as cell-penetrating moiety; (iii) Cys_KLA was more effective than KLAR7, a globally recognized CPP; (iv) The combination of Cys and R7 as cell penetration moieties for KLA pro-apoptotic peptide was effective for enhancing the overall cytotoxic effect; (v) The cytotoxic responses were dependent of the time of incubation and concentration of KLA moieties; the higher the incubation time and the concentration, the higher was the cytotoxicity; (vi) Another key aspect was observed regarding the relative “protective effect” of the nanoconjugates towards the HEK 293T compared to cancer cells (U-87 MG). The set of properties featured in these nanoconjugates are highly desired for preventing and minimizing the collateral side-effect of chemotherapeutic drugs while preserving an efficient targeted cellular delivery. These are key issues that have hampered the progress of anticancer therapy, relying on the development of novel nanomedicines comprising polymer-drug conjugation, biomolecules, and hybrid nanostructures to overcome the challenges.

**Figure 7:**
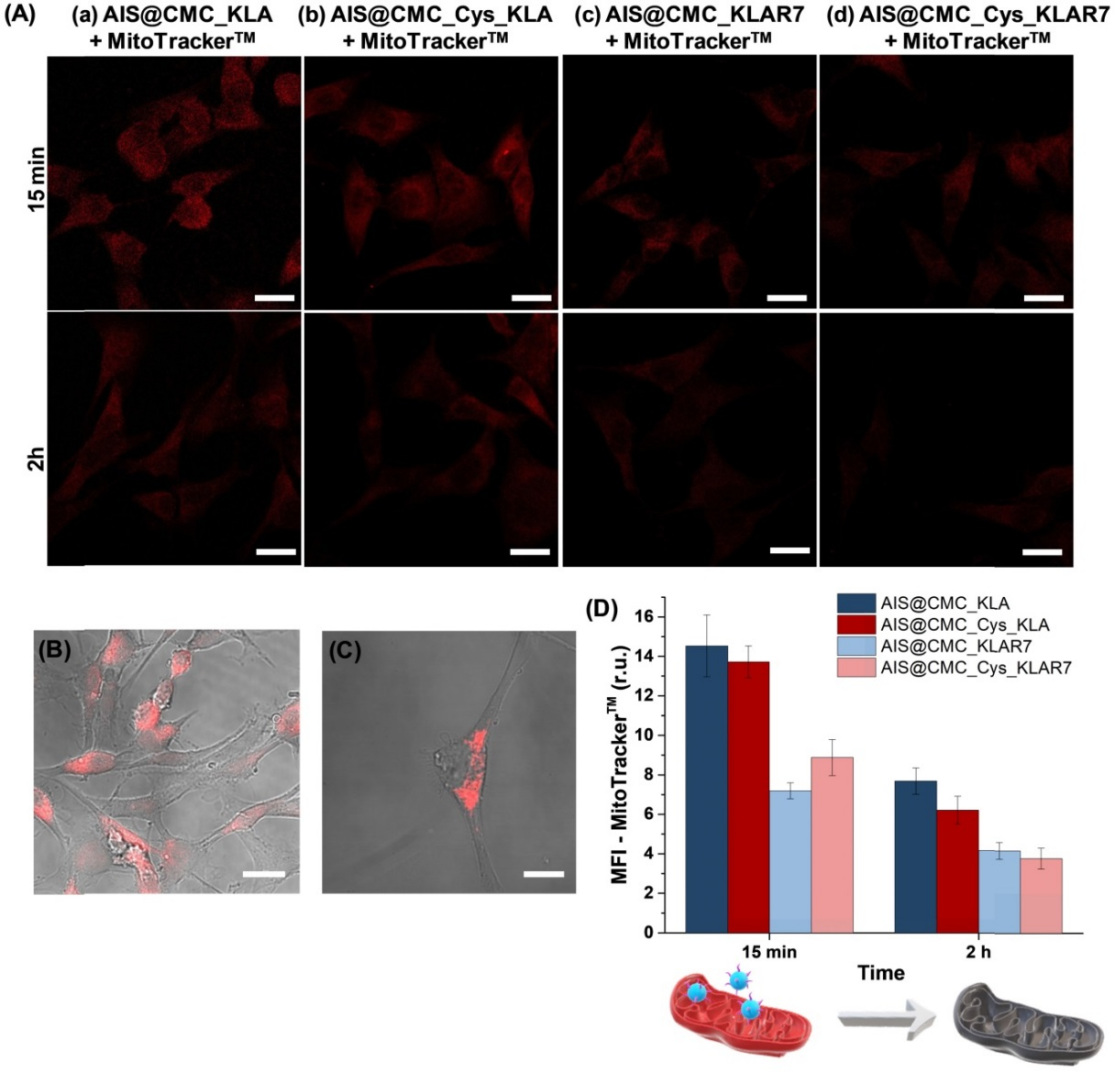
(A) CLSM images of U-87 MG cells incubated with (a) AIS@CMC_KLA, (b) AIS@CMC_Cys_KLA (c) AIS@CMC_KLAR7, and (d) AIS@CMC_Cys_KLAR7 for 15 min and 2 h for evaluation of red emission of MitoTracker™ (TRITC channel). Fluorescence images of (B) reference sample (cell stained with MitoTracker™ without nanoconjugates) and (C) control sample (cell stained with MitoTracker™ after 2 h of incubation with AIS@CMC sample) (scale bar = 10 μm). (D) MFI plot of red signals after 15 min and 2 h of incubation with bioconjugates (mean ± standard error (SE); n = 6 cells). Refer to Figure S9 for autofluorescence control images and reference images (“blanks”) of AIS@CMC-based and AIS@CMC_Cys-based nanoconjugates at the red channel.

**Mitochondria Staining (MitoTracker™ biomarker)**. In addition to the MTT test, the evaluation of *in vitro* toxicity of nanoconjugates was performed using MitoTracker™ biomarker for mitochondria staining, considering that the cell-killing mechanism of KLA-based therapeutic peptides involved the mitochondrial membrane disruption. Thus, as this fluorophore (MitoTracker™) is concentrated by active mitochondria, it was used as a tool for tracking the action of KLA and KLAR7-modified nanoconjugates. Therefore, U-87 MG cells were incubated with the bioconjugates for 15 min and 2 h, and in the sequence, labeled using MitoTracker^TM^ Deep Red FM for evaluation using confocal laser scanning microscopy (CLSM) (Figure 7A). As a general trend, the fluorescence images evidenced a significant reduction of the red emission with time, confirming mitochondria damage by KLA and KLAR7 peptides conjugated to both AIS@CMC and AIS@CMC_Cys nanohybrids. The thiolated nanostructures (Figure 7A(b) and Figure 7A(d)) resulted in a higher reduction of Mitotracker™ fluorescence emission in comparison to the corresponding nanoconjugate without L-cysteine (Figure 7A(a) and Figure 7A(c)). These results supported the MTT assays in previous sections and validated the crucial role of the presence of cysteine in cell uptake by U-87 MG cells. Moreover, it is also observed that the cells treated with R7-containing nanoconjugates (Figure 7A(c-d)) underwent a faster reduction of red signals, with the lowest emission observed for sample with two penetrating moieties (Cys and R7) and KLA cell death agent after 2 h.

As the control sample and to confirm that the reduction of MitoTracker™ signals through time was mostly related to the presence of the KLA peptide grafted to the nanoconjugates and not associated with photobleaching, the bare AIS@CMC sample was also incubated with U-87 MG cells for 2 h, and stained with MitoTracker™. The CLSM image (Figure 7C) was compared with the reference samples (*i.e.,* U-87 MG cell labeled with MitoTracker™ and without conjugates, Figure 7B). Opposite to the observations of KLA-modified nanoconjugates, no significant reduction of MitoTracker™ fluorescence was observed after 2 h of incubation with AIS@CMC nanoconjugate. All trends perceived by the qualitative analyses of CLSM images were confirmed by the measurements of mean fluorescence intensity (MFI, Figure 7D) obtained through image processing software (public domain, ImageJ, v.1.5+).

Interestingly, these results of MTT and mitochondria staining revealed that the presence of the R7 CPP was active but not essential for the cell-killing effect of KLA, opposing other systems reported 16. These findings proved that AIS@CMC and AIS@CMC_Cys can act as bifunctional nanocarriers for the delivery of therapeutic peptides (*i.e.,* both CMC and CMC_Cys as cell-penetrating agents) as well as tracking intracellular pathways (AIS as inorganic fluorescent nanoprobe). Thus, in other to explore this avenue of new possibilities as active cell-penetrating agents, CMC and CMC-Cys-bearing nanoconjugates coupled to KLA were further investigated in the next sections because the effects of R7-CPP sequences have been well-characterized in the literature.

**Figure 8:**
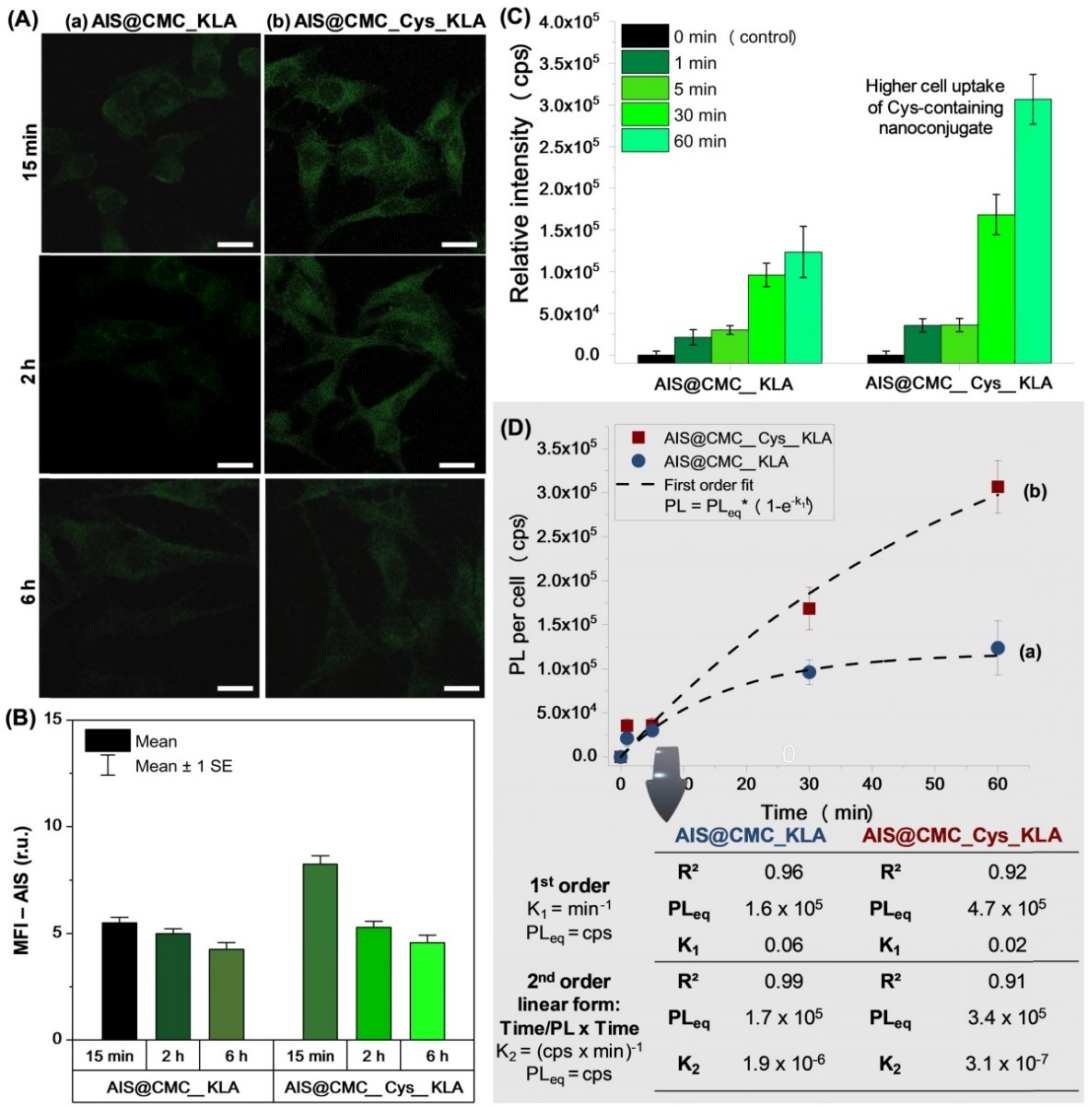
(A) CLSM images of cellular uptake of (a) AIS@CMC_KLA and (b) AIS@CMC_Cys_KLA bioconjugates incubated with U-87 MG cells for 15 min, 2 h, and 6 h. (scale bar = 10 μm) and corresponding (B) MFI values for AIS QDs emission (mean ± SE; n ≥ 6 cells). (C) Accumulation of AIS@CMC_KLA and AIS@CMC_Cys _KLA in U-87 MG cells (λ_excitation_ = 360 nm) with time. (D) Results of pseudo-first order and pseudo-second-order kinetics modeling for cellular uptake of (a) AIS@CMC_Cys_KLA, and (b) AIS@CMC_KLA. (mean ± SD; n = 3).

**Cellular uptake of QD nanoconjugates by CLSM and steady-state fluorescence.** As the results of previous sections demonstrated that the increase of cell uptake of thiolated peptidomimetic nanoassemblies was responsible for the higher lethality verified, the photoluminescent properties of AIS QDs were used for tracking of cell uptake of AIS@CMC_Cys_KLA in comparison to AIS@CMC_KLA using CLSM (Figure 8). Although AIS quantum dots and nanoconjugates possess the main peaks of red emission (Figures 2B and 5B), at the intracellular medium, the fluorescent signals of cells treated with nanoconjugates were detected in green color (FITC filter) without detectable emissions in red (TRITC filter, Figure S9). This effect can be associated with the high Stokes shift frequently observed for these ternary quantum dots, which affected the matching of excitation/emission properties of the AIS QDs with the available fluorescence microscope filter cubes. Moreover, the CLSM images comprise the overall combination of the characterization method with the effects of the intracellular microenvironment (*e.g.,* protein corona) on the optical properties of AIS QDs QDs (fluorescent shift to a shorter wavelength) as reported in the literature 76,77.

For all of the times and samples evaluated, the typical green fluorescent signals of AIS QDs 23 were detected scattered in the cytoplasm but not inside the cell nucleus (Figure 8A). Based on the images and MFI values (Figure 8B), it was observed a higher intensity of fluorescence emission for the AIS@CMC_Cys_KLA sample (Figure 8A(b)) compared to the sample without L-cysteine (Figure 8A(a)) after incubation (15 min, 2 h, and 6 h) of bioconjugates with U-87 MG cell line, confirming the rational design of Cys as cell-penetrating moiety. After 6 h of treatment, a reduction of the emission signals was verified for both samples, which can be associated with the exocytosis process as well as the reduction of the concentration gradient between intra and extracellular media as time evolved.

Moreover, steady-state fluorescence spectroscopy was used to evaluate the internalization dynamics of the bioconjugates based on the fluorescence of the inorganic core of AIS QDs. The PL emission plot based on the intensity per cell for AIS@CMC_KLA and AIS@CMC_Cys_KLA is depicted in Figure 8C and showed that the intracellular uptake of both nanoassemblies increased with the incubation time. For assessing the kinetics of cell uptake, the data were fitted to first (coefficient of determination, R^2^ ≥ 0.92), and second-order (R^2^ ≥ 0.91) kinetic models. Based on the results (Figure 8D), the uptake of AIS@CMC_Cys_KLA by U-87 MG cells occurred at a relatively higher rate and superior PL of equilibrium (PL_eq_) when compared to that of AIS@CMC_KLA. Additionally, for the AIS@CMC_Cys_KLA sample (Figure 8D(b)), the absence of a plateau in the kinetics curve suggests that the equilibrium was not achieved within the time interval evaluated (*i.e.,* up to 60 min of incubation). It indicates that a further increase in the level of uptake is expected to occur for longer incubation times with these nanoconjugates, as predicted by the calculated values of PL_eq_. Despite the fact that multiple events are taking place simultaneously, involving the internalization of the nanoconjugates, these results offer solid evidence that grafting the Cys residues to the CMC macromolecular structure boosted the delivery of these designed peptidomimetic targeted nanoconjugates into U-87 MG cells.

**Colocalization of KLA peptide with mitochondria.** Most drug delivery systems based on cell-penetrating moieties enter the cytoplasm by endocytosis, where the delivery cargo can be captured by endosomes and lysosomes 78. It has been broadly reported that amide linkages present in peptides and also used in this study for pro-peptide conjugation is one of the most stable bonds in the intracellular environment. Therefore, although viable, amide bonds are not necessarily broken into the endo-lysosome compartment through enzyme-mediated catalysis 22. In fact, the reaction kinetics is slow and is likely not to have affected the capabilities of the nanoconjugates for simultaneous bioimaging and mitochondrial dysfunction.

**Figure 9:**
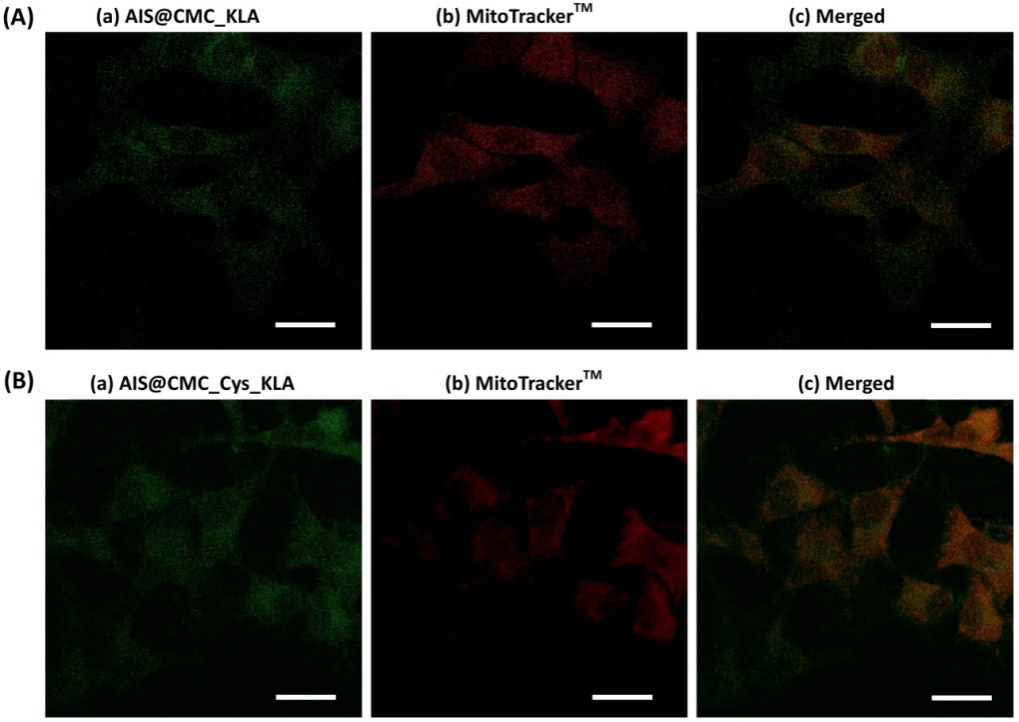
CLSM images of U-87 MG cells incubated for 2 h with (A) AIS@CMC_KLA and (B) AIS@CMC_Cys_KLA analyzed for the evaluation of the (c) colocalization (merged image) of (a) green signals of nanoconjugate and (b) red MitoTracker™ signals (scale bar = 10 µm).

In order to follow the fate of AIS@CMC_KLA (Figure 9A) and AIS@CMC_Cys_KLA (Figure 9B) upon internalization, the intracellular distribution of fluorescent nanoconjugate and colocalization with mitochondria was analyzed using CLSM. The merged images (Figure 9c) from AIS (Figure 9a) and MitoTracker^TM^ (Figure 9b) indicated regions with colocalized signals (yellow) associated with the green signals of AIS QDs used for tracking KLA peptide overlapped with the red fluorescence of the targeted mitochondrial network. Also, a subpopulation of *pure* green pixels was observed well-dispersed throughout the other regions of cytoplasm associated with the intracellular trafficking of nanoconjugates before and after interaction with mitochondria. The Manders' overlap coefficient was the colocalization indicator calculated using image processing software (JaCoP plugin, ImageJ) for the incubation time of 2 h of the nanoconjugates with U-87 MG cells. This coefficient ranges from 0 to 1, with 0 indicating non-overlapping images and 1 reflecting 100% colocalization between both channels 79. The degree of colocalization of nanoconjugates and mitochondria signals was 0.35 ± 0.04 for AIS@CMC_KLA (moderate degree) and 0.52 ± 0.04 for AIS@CMC_Cys_KLA (high degree). These results indicated that KLA conjugates interacted with the mitochondria membrane and performed their pro-apoptotic function coupled to the fluorescent nanocarriers (AIS@CMC and AIS@CMC_Cys), without requiring the rupture of amide bonds. Moreover, the values of the Manders' overlap coefficient were in agreement with the results of cell death (MTT and the reduction of MitoTracker^TM^ signals), indicating the higher efficiency of CMC-Cys-modified nanoconjugates.

**Nanocarriers for dual-targeting therapy - AIS@CMC-based pro-apoptotic nanoconjugates coupled to the chemotherapeutic anticancer drug (DOX)**. The combination of polymers and drugs has emerged as a powerful weapon for fighting life-threatening diseases motivated by the possibility of producing polymer-drug conjugates with designed architectures and chemical functionalities with bioactivity towards damaged tissues 80. Thus, based on the aforementioned results and discussed in the previous sections, we have designed and produced bi-functional QD-macromolecule bioconjugates, which demonstrated to act effectively as fluorescent nanoprobes as well as pro-apoptotic nanosystems. Hereafter, we amalgamated these developed bioconjugates comprising the AIS@CMC-peptides with the anticancer drug DOX, forming newly hybrid polymeric prodrug nanocarriers.

To this end, DOX was explored as a model chemotherapeutic drug for comparing the effect with the KLA-modified nanoconjugates as well as at combining both systems for prospective synergic enhanced therapy against glioblastoma cells based on a novel dual-targeted therapy. That means KLA and DOX were utilized as mitochondria-targeting and nucleus/DNA-targeting killing agents, respectively.

**Figure 10:**
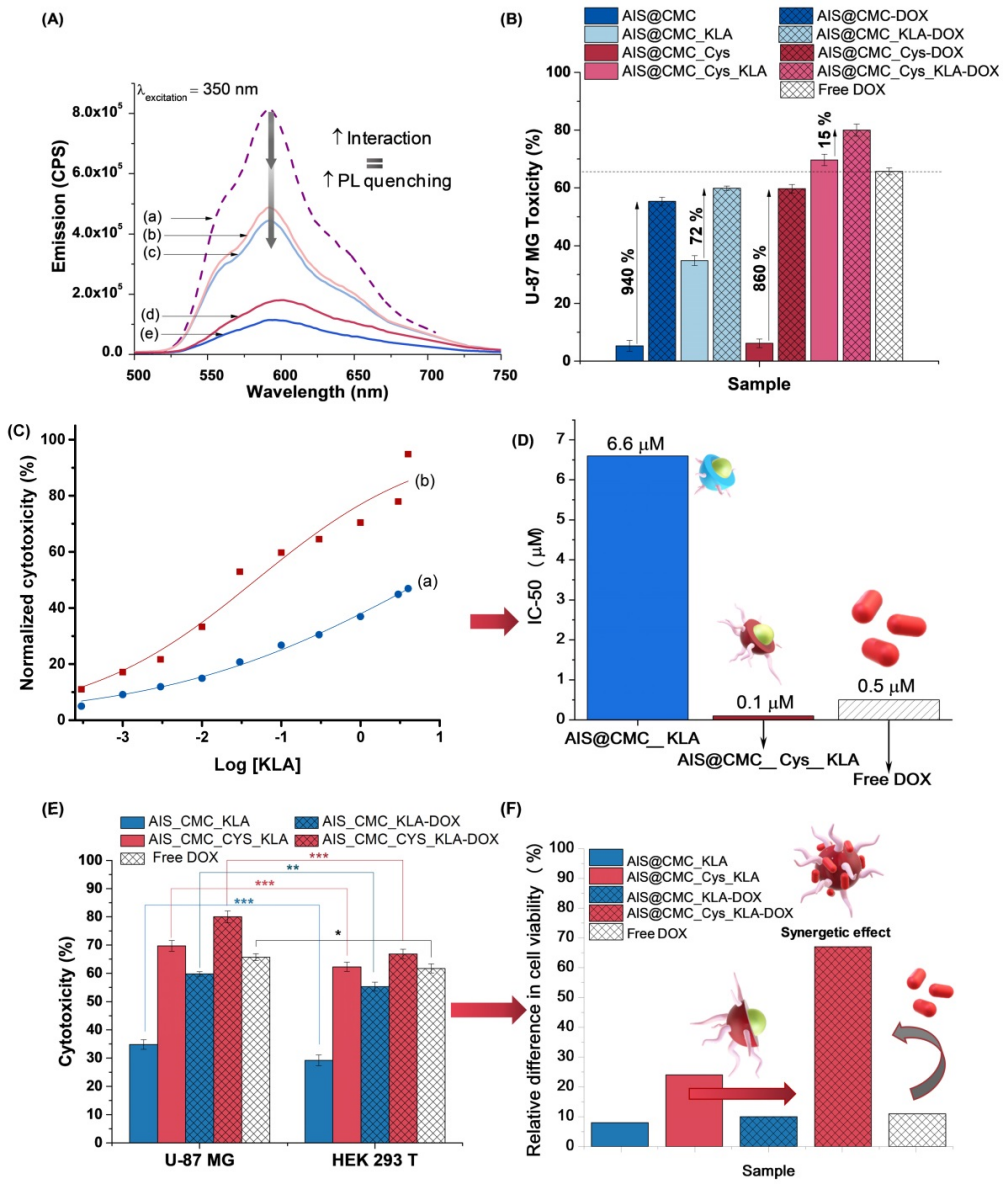
(A) PL emission spectra of (a) Free DOX, (b) AIS@CMC_Cys _KLA-DOX, (c) AIS@CMC_KLA-DOX, (d) AIS@CMC_Cys-DOX, and (e) AIS@CMC-DOX. (B) MTT results for conjugates and nanocarriers after incubation for 24 h with U-87 MG cell line. (C) Normalized dose-response curves for (a) AIS@CMC_KLA and (b) AIS@CMC_Cys_KLA. (D) Half maximal effective concentration (EC-50) results for bioconjugates in comparison to free DOX 62. (E) Comparison of toxicity of U-87 MG and HEK 293T after 24 h of incubation with bioconjugates and nanocarriers (mean ± standard deviation (SD); n = 6; One-way ANOVA followed by Bonferroni's test with *: p ≤ 0.05, **: p ≤ 0.01, and ***: p ≤ 0.001). (F) Increase in cell viability for normal cells compared to glioblastoma cell line.

The nanocarriers (AIS@CMC-DOX, AIS@CMC_KLA-DOX, AIS@CMC_Cys-DOX, and AIS@CMC_Cys_KLA-DOX) were obtained by the complexation of cationic DOX and negatively charged nanoconjugates (Table 3). PL spectra of all samples were collected to demonstrate the interactions of the drug with the nanoconjugates leading to the formation of hybrid prodrug nanostructures, which was confirmed through the quenched emission of the drug-loaded nanocarriers compared to the emission of “free” DOX (Figure 10A). The quenching of DOX emission (Figure 10A(a)) was considerably higher when associated with AIS@CMC nanoconjugate (Figure 10A(e)), which possesses a more negative surface charge (ζ = - 49 mV) and the smaller hydrodynamic diameter (D_H_ = 74 nm). On the contrary, the combination AIS@CMC_Cys_KLA with DOX (Figure 10A(b)), which involves the larger (D_H_ = 139 nm) and less charged nanoconjugate (ζ = - 13 mV), suffered a minor reduction of the PL intensity compared to the free DOX. These results are consistent with the mechanisms of quenching of DOX orange-red emission that is mostly associated with electrostatic interactions between DOX-NH_3_^+^ --- COO^-^ groups, in addition to hydrophilic and hydrophobic interactions, π-π stacking and self-assembly 1.

To further investigate the effect of these designed nanostructure complexes on the mitochondria activity, the MTT assay was performed with U-87 MG (Figure 10B) and HEK 293T (Figure S10) cell lines where the relative cytotoxicity was evaluated for the free DOX, nanoconjugates (AIS@CMC, AIS@CMC_KLA, AIS@CMC_Cys, and AIS@CMC_Cys_KLA) and nanocarriers (nanoconjugates + DOX). For comparison purposes, the final concentration of KLA and/or DOX killing agents was 4 µM in each well. It was observed that the complexation with DOX of all of the nanoconjugates increased the lethality attributed to the chemotherapeutic action of the drug. Obviously, the highest increase in cytotoxicity was observed for AIS@CMC and AIS@CMC_Cys complexed with DOX compared to the analogous pristine conjugates. This was expected, as the original nanoconjugates were not cytotoxic before the incorporation of DOX, which acted as the cell-killing agent (*i.e.,* payload release). Albeit, these nanocarriers presented lower toxicity compared to the free drug. This effect can be assigned to the electrostatic interactions of positively charged DOX molecules with anionic conjugates forming complexes that require being broken for releasing the active payload to enter the cell nucleus to trigger apoptosis by intercalation with DNA. In this sense, the complexation with nanocarriers modulated the kinetics of DOX release (dependent on conjugate surface charge and internalization rate), circumventing the burst effect of the drug but without hampering its anti-cancer activity. The relative higher toxicity of AIS@CMC_Cys-DOX to cells in comparison to AIS@CMC-DOX was explained based on the weaker interactions between conjugate to the drug (less negative) joined with the already verified enhanced cellular internalization of thiol-bearing nanoconjugates.

The sample AIS@CMC_KLA-DOX combining two killing agents (*i.e.,* KLA and DOX) was less lethal than free DOX for both U-87 MG (Figure 10B) and HEK 293T (Figure S10) cell lines, although higher than AIS@CMC_KLA conjugates. Conversely, AIS@CMC_Cys_KLA bioconjugate was remarkably more effective in killing cells than free DOX, the anti-cancer model drug. This is a fascinating result when considering the common side effects of DOX in cancer chemotherapy to non-targeted tissues (predominantly heart), which restricts the correct dosage of the drug and reduce the quality of life of patients under clinical treatment with DOX 81. In addition, confirming the rational design of this study, the amalgamation of AIS@CMC_Cys_KLA with DOX (AIS@CMC_Cys_KLA-DOX) formed the most lethal nanoconjugates against the brain cancer cells. This effect was ascribed to the combined mechanisms of death induction promoted by KLA, causing mitochondria disruption, and by DOX targeting DNA at the cell nucleus. Moreover, this tendency was enhanced by grafting of L-cysteine (Cys) to the macromolecular structure as cell-penetrating moiety, increasing the cellular uptake of the nanocarrier.

**Figure 11:**
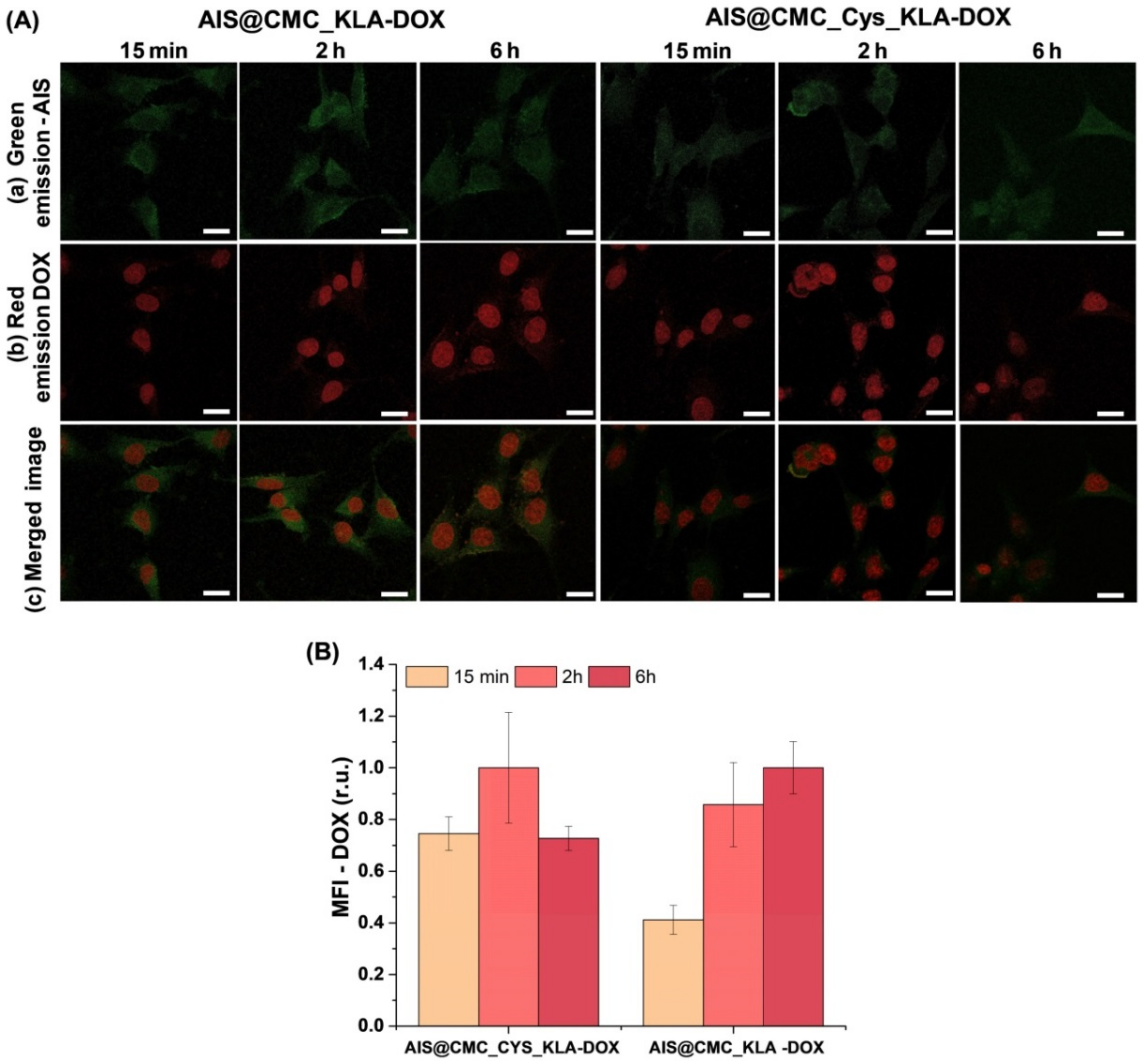
(A) Single channel ((a) FITC and (b) TRITC filters) and merged images (c) of CLSM for 15 min, 2 h, and 6 h incubation of AIS@CMC_KLA-DOX and AIS@CMC_Cys_KLA-DOX with U-87 MG cells (scale bar = 10 μm). (B) MFI results for DOX signals after 15 min, 2 h, and 6 h of incubation of U-87 MG cells with nanocarriers (mean ± SE; n ≥ 6 cells).

CLSM analyses were performed to track these effects of the combined therapy (*i.e.,* KLA-modified hybrid nanoconjugates and DOX) using U-87 MG cells incubated with AIS@CMC_KLA-DOX and AIS@CMC_Cys_KLA-DOX (Figure 11A). For both nanocarriers, the green emission of AIS QDs was detected scattered in the cytoplasm (Figure 11A(a)), and the red signals of DOX inherent fluorescence (Figure 11A(b)) were observed mostly in the cell nucleus since 15 min of incubation, confirming the rapid permeation and internalization of the hybrid pro-drug nanosystems in the cells. Additionally, MFI measurements of the DOX signals (red fluorescence) inside U-87 MG cells revealed a higher intensity of emission at shorter periods of incubation for the thiomer-based nanocarrier (Figure 11B), in agreement with CPP-action of Cys residues. Finally, the reduction of the DOX signals for AIS@CMC_Cys_KLA-DOX after 6 h of incubation is associated with quenching of drug emission after intercalation with DNA 82.

Then, MTT *in vitro* results indicated a prospective future clinical therapeutic application of AIS@CMC_KLA and AIS@CMC_Cys_KLA nanoconjugates for brain cancer treatment as substitutes to DOX, which is known to trigger severe side-effects when systemically administrated in chemotherapy. Thus, the half-maximal effective concentrations (EC-50) of both samples were calculated (Figure 10D) for U-87 MG cell lines incubated for 24 h with different concentrations of the bioconjugates. From dose-response curves (Figure 10C) fitted using the general equation for a sigmoidal model (Normalized toxicity *versus* log(KLA) concentration, R^2^ > 0.96) 83, KLA peptide attached to the non-toxic AIS@CMC conjugate achieved an EC-50 value as low as 6.6 μM. Remarkably, the presence of Cys residues in AIS@CMC_Cys_KLA promoted an EC-50 at submicromolar concentrations of the KLA peptides (0.1 μM), approximately 70-fold smaller in comparison to AIS@CMC_KLA. According to the literature 3,28,29, KLA peptide alone presents EC-50 values typically > 100 μM, while in the presence of CPP moieties (*e.g.,* R7), the EC-50 can vary from 3 to 25 μM depending on the cancer cell line. The obtained EC-50 result for AIS@CMC_KLA (6.6 μM) confirmed that the CMC polymer favored the internalization of KLA. Outstandingly, the EC-50 for AIS@CMC_Cys_KLA was radically smaller (from 30-fold to 250-fold) compared to those of previously reported KLA-based anticancer therapeutics 3,11,28,29,84,85.Moreover, regarding potential anticancer therapy, this value is approximately 5-fold lower than common values reported for regular clinical applications of the model drug doxorubicin (~ 0.5 μM for U-87 MG cells) 86. Thus, the achievement of such a low EC-50 value presented in this work is outstanding for the development of new anticancer agents once it may allow much lower therapeutic dosages while retaining the killing effectivity of cancer cells. Consequently, these smart nanoconjugates would prevent the widely known negative collateral effects associated with high dosages of anticancer drugs that are frequently required in the conventional treatment of resistant tumors 8.

It is important to emphasize that all of the bioconjugates and nanocarriers (*i.e.,* bioconjugates + DOX) assayed presented a slight cell selectivity (“selectivity index” of 1.1-1.2) towards the glioblastoma cells compared to the normal HEK 293T cell line (Figure 10E). It was observed as a relative reduction of cytotoxicity towards healthy cells compared to tumor cells. For instance, AIS@CMC_Cys_KLA-DOX nanocarriers revealed a substantial enhancement of approximately 67% of cell viability (100% - toxicity) for HEK 293T compared to U-87 MG cells (Figure 10F). Hence, these innovative AIS@CMC_Cys_KLA-DOX nanocarriers amalgamated extremely important features in cancer therapy, high lethality, low drug concentration, and relative specificity towards tumor cells. Moreover, due to the presence of the fluorescent inorganic core, they also acted as an active nanoprobe for cellular bioimaging and tracking, which offers alternatives in cancer nanotheranostic applications. Although extremely motivating, clearly, these findings cannot the straightforwardly applied to the clinical use of the novel nanoconjugates in brain tumor therapy, where the ability to cross the blood-brain barrier (BBB), the circulation time in the blood, and other relevant aspects are required to be properly addressed in future research.

**Figure 12:**
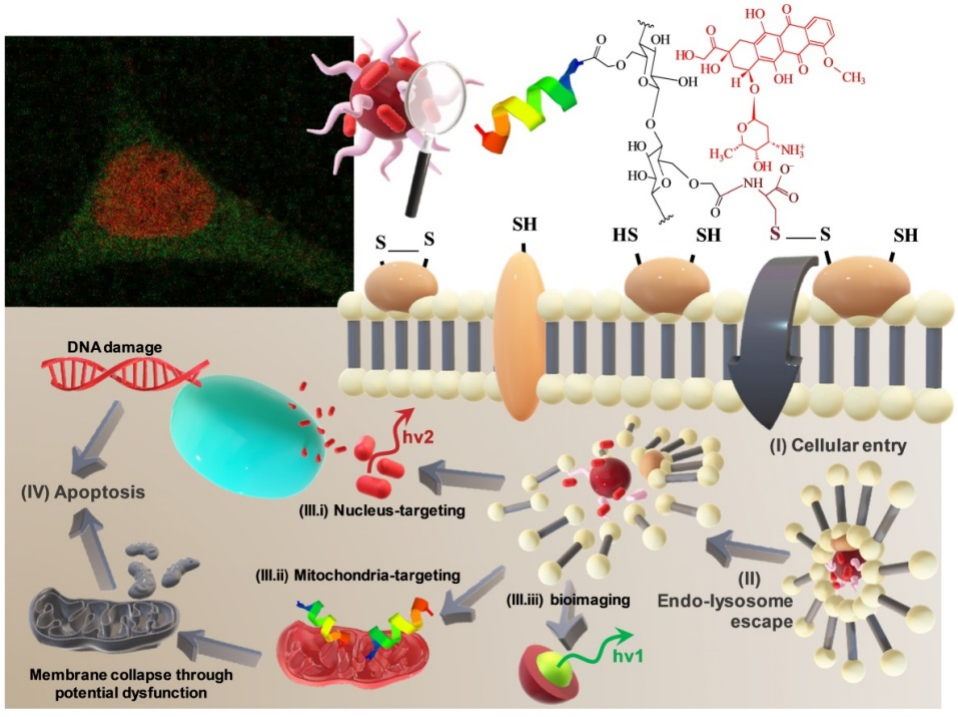
Schematic illustration of the multifunctional behavior of hybrid nanosystems composed of AIS@CMC_Cys_KLA-DOX for targeted therapy of brain cancer cells *in vitro* (not to scale).

To this end, Figure 12 summarizes the schematic representation of the two integrated mechanisms developed in this study that induced the deaths of the brain cancer cells *in vitro* promoted by KLA peptides and DOX drug, constructed through bioengineered macromolecular hybrid nanosystems complexed with DOX.

## Conclusions

In summary, we rationally designed and developed novel hybrid nanosystems through a *green* chemistry process combined with a nanotheranostic strategy for targeted anticancer therapy applications. These nanohybrids encompassed a fluorescent semiconductor core of Ag-In-S quantum dot (AIS QD) with chemically modified carboxymethyl cellulose (CMC) as polysaccharide-based macromolecular ligands. They were biofunctionalized with L-cysteine amino acid (Cys), R7 (arginine-rich model cell-penetrating peptide, CPP), and KLA peptides (pro-apoptotic payload for mitochondria disruption). These nanoconjugates were comprehensively characterized through morphological and spectroscopic techniques, which demonstrated the chemical conjugation with the biological modifiers (*i.e.,* Cys, R7, and KLA) by the formation of amide bonds with CMC, rendering the development of supramolecular nanoarchitectures. Moreover, the nanoconjugates presented surface chemistry and morphological features dependent on the biofunctionalization associated with photoluminescent optical activity suitable for cell bioimaging. *In vitro* cytotoxicity evaluation of the nanoconjugates using MTT mitochondrial activity revealed that AIS@CMC and AIS@CMC_Cys were non-toxic. Additionally, the cysteine-bearing (-Cys) nanoconjugates demonstrated an important enhancement of the cell-penetrating activity comparable to the model arginine-rich modifier (-R7), which was ascribed to the cysteine-mediated internalization through interactions with the thiol-rich domains at cell membranes. Moreover, the grafting of cysteine combined with KLA peptides was highly effective in increasing the cellular internalization of bioconjugates (AIS@CMC_Cys_KLA), which drastically boosted their killing activity of cancer cells. This remarkable effect was ascribed to the amalgamation of the cell-penetration capability of Cys with cell apoptosis driven by KLA in directing mitochondrial dysfunction. Notably, these findings demonstrated that the Cys-bearing nanoconjugates associated with KLA were more effective as bifunctional agents for killing cancer cells than the analog nanoassemblies modified with a well-known model CPP modifier (R7). But even more striking was the result showing that the relative lethality of these novel nanoconjugates was cell-type dependent, where it was less pronounced in the healthy cells compared to the glioma tumor cells, by comparing the cell deaths induced by the same bioconjugate nanosystem. Hence, these results demonstrated that the bioconjugates showed relative cell selectivity towards cancerous cells, which was assigned to the presence of thiol-rich receptor domains overexpressed by glioblastoma cells. This preliminary slight “protective effect” observed for these nanoconjugates towards normal cells is a highly desirable feature as the ultimate goal of cancer therapy relies on eliminating malignant tumors while preserving healthy tissues. Furthermore, bearing in mind the prospective application on cancer therapy, these nanoassemblies were tested as nanocarriers through *in vitro* cytotoxicity assays for simultaneously delivering DOX chemotherapeutic drug. The AIS@CMC_Cys_KLA bioconjugate was extraordinarily more effective in killing brain cancer cells than “free” DOX, considered the anti-cancer model drug. This is a key result when considering the common side-effects of DOX observed in conventional cancer chemotherapy. Confirming the rational design of this study, the amalgamation of AIS@CMC_Cys_KLA with DOX (AIS@CMC_Cys_KLA + DOX) formed the most lethal nanoconjugates against the cancer cells. This effect was credited to the synergic behavior combining the mechanisms of death induction promoted by KLA as a targeted organelle agent provoking mitochondria disruption and by DOX drug targeting DNA dysfunction at cell nucleus. To conclude, we envision that these findings can pave the way for developing novel nanoplatforms applied for simultaneously imaging and bimodal targeted-therapy (KLA mitochondria-targeted and DOX nucleus-targeting) against brain cancer while protecting healthy tissues and minimizing the potential high systemic toxicity and side-effects of conventional anti-cancer chemotherapy.

## Methods

### Materials and cell lines

Carboxymethylcellulose sodium salt (CMC, DS = 0.7, M_w_ = 250 kDa; viscosity = 735 cps at 2% in H_2_O, at 25 ºC), L-cysteine hydrochloride (Cys, HSCH_2_CH (NH_2_)COOH · HCl, ≥ 98%), and doxorubicin hydrochloride (DOX, C_27_H_29_NO_11_ ⋅ HCl, ≥ 98.0%) were purchased from Sigma-Aldrich (USA). KLA (LAKLAKKLAKLAK) and KLAR7 (RRRRRRRKLAKLAKKLAKLAK) peptides were purchased from GenScript (USA). All of the other materials used in this research were detailed in the Supplementary Material.

Human embryonic kidney cells (HEK 293T, American Type Culture Collection - ATCC CRL-1573) were provided by the Federal University of Minas Gerais/UFMG. Human brain likely glioblastoma cells (U-87 MG, ATCC HTB-14) were supplied from Brazilian Cell Repository (Banco de Células do Rio de Janeiro: BCRJ, Brazil; cell line authentication molecular technique, Short Tandem Repeat (STR) DNA; quality assurance validation by international standard NBR ISO/IEC 17025:2005).

### Synthesis of AIS@CMC quantum dots and thiomers

The synthesis of AIS@CMC, as well as the functionalization of AIS@CMC with L-cysteine, were fully described in previously published work 23 and can be found in Supplementary Material.

### Coupling of KLA and KLAR7 peptides to AIS@CMC and AIS@CMC_Cys conjugates

**Figure 13:**
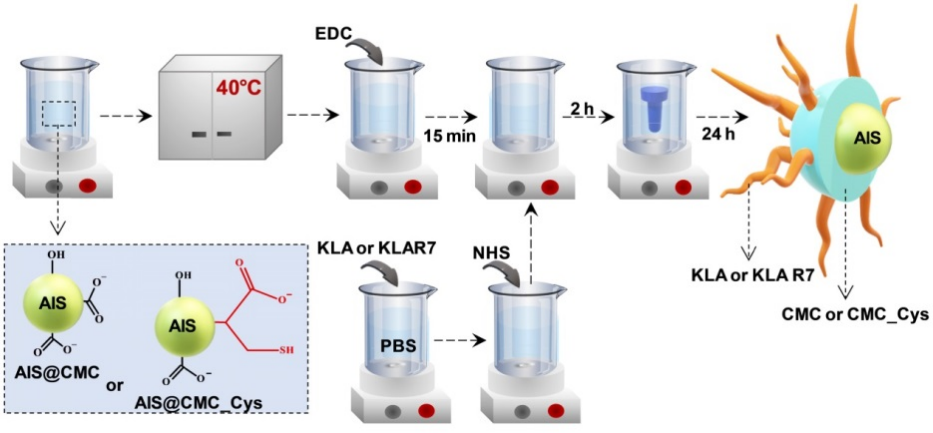
Schematic representation of the synthesis of bioconjugates.

The coupling of the therapeutic peptides (KLA and KLAR7) to AIS@CMC or AIS@CMC_Cys conjugates was performed in aqueous media by amide bond formation using EDC and N-hydroxysulfosuccinimide sodium salt (sulfo-NHS) chemistry. Briefly, 10 mL of AIS@CMC or AIS@CMC_Cys suspensions previously prepared were dried in an oven using hot air at 40 ± 1 ^º^C to achieve a volume of 5 mL by solvent evaporation. Then, 0.2 mL of distilled water containing 3.8 mg of EDC were poured into each colloidal QD suspension and stirred for 15 min for the activation of carboxylate groups of the polymer. Meanwhile, solutions of KLA or KLAR7 (5 mL) were prepared by dissolving the peptides in phosphate buffer saline (PBS, 2x, pH 7.4 ± 0.1) in molar ratios of 1:4 or 1:20 (peptide:COO^-^, considering the content of carboxylates of CMC in 10 mL of QDs colloidal suspensions before concentration) and 10.9 mg of sulfo-NHS were added to each peptide solution. In the sequence, the peptide/sulfo-NHS solution was poured into carboxylate-activated QD suspensions and incubated for 2 h in an ice bath for producing the bioconjugates (AIS@CMC_KLA, AIS@CMC_KLAR7, AIS@CMC_Cys_KLA, and AIS@CMC_Cys_KLAR7). After this period, 1.5-fold excess of ethanolamine hydrochloride (ETA) related to EDC was introduced into the flasks and stirred for 10 min to stop the reaction. All of the bioconjugates were dialyzed against 4 L of distilled water (pH = 8.0 ± 0.1, adjusted with NaOH 1 M) for 24 h with two dialysate changes (Pur-A-Lyzer™Mega Dialysis Kit, cellulose membrane, 12 kDa cut-off, Sigma-Aldrich). In the sequence, bioconjugate suspensions were dried (40 ± 1 ^º^C) to reduce the final volume to 10 mL by solvent evaporation. Figure 13 summarizes the functionalization steps of AIS@CMC and AIS@CMC_Cys quantum dots with KLA and KLA7 peptides.

### Coupling of AIS@CMC-based nanoconjugates with doxorubicin (DOX)

Prodrugs were obtained by electrostatic interactions between negative carboxylate groups from conjugates and cationic doxorubicin (DOX). Nanocarriers (AIS@CMC-DOX, AIS@CMC_Cys-DOX, AIS@CMC_KLA-DOX, and AIS@CMC_Cys_KLA-DOX) were prepared by dropping under stirring, 64 μL of DOX solution (10 mM) into the reaction flask with 4 mL of each nanoconjugate colloidal solution (or 4 mL of DI water for Free DOX reference solution), yielding a molar ratio of DOX:COO^-^ (from CMC) of approximately 1:8.5. All solutions were homogenized for 1 h under vigorous magnetic stirring in an ice bath while covered to avoid light exposure.

### Physicochemical, morphological and spectroscopic characterization of AIS@CMC -based conjugates

Quantum dots, thiomers, peptidomimetic nanoassemblies, and nanocarriers were extensively characterized by several techniques. All experimental procedures were detailed in the Supplementary Material.

Optical properties of conjugates were obtained using ultraviolet-visible spectroscopy (UV-vis, transmission mode, Lambda EZ-210, PerkinElmer) and photoluminescence spectroscopy (steady-state: λ_excitation_ = 350 nm; and TCSP: DeltaDiode - pulsed laser, λ_excitation_ = 375 ± 10 nm, λ_emission_ = 625 nm. FluoroMax-Plus-CP, Horiba Scientific). Quantum yield (QY) values were estimated using the comparative method using Rhodamine 6G (Sigma-Aldrich) at λ_excitation_ = 488 nm.

Morphological features were assessed from transmission electron microscopy (TEM, 200 kV, Tecnai G2- 20-FEI, FEI Company) and atomic force microscopy (AFM, mode = non-contact tapping, frequency = 325 kHz, scanning rate = 1.0 Hz, pixel resolution = 512 × 512, XE-70, Parker Systems) images.

Energy-dispersive x-ray spectroscopy (EDX, EDAX detector coupled to Tecnai G2- 20-FEI) and X-ray photoelectron spectroscopy (XPS, excitation source = Mg-Kα, Amicus, Kratos Analytical) techniques were used for evaluating elemental composition and oxidation states of AIS QDs.

The chemical functionalization of AIS@CMC with Cys, KLA, and KLAR7 was investigated using Fourier-transform infrared spectroscopy (FTIR, mode = attenuated total reflectance (ATR), range = 4000-850 cm^-1^, scans = 32, resolution = 4 cm^-1^ resolution, Nicolet 6700, Thermo-Fischer), proton nuclear resonance spectroscopy (^1^H-NMR, frequency = 400 MHz, scans = 64, Avance™III DH NanoBay, Bruker), dynamic light scattering (DLS, 35 mW red diode laser λ = 660 nm, ZetaPlus, Brookhaven Instruments Corporation) and zeta potential (ζ, ZetaPlus, Brookhaven Instruments Corporation) analyses. In addition, Ellman's method was used to determine the degree of functionalization of AIS@CMC_Cys with thiol groups (Figure S11) 87.

### Biological experiments

The detailed protocols of all the biological tests were presented in Supplementary Material.

#### Evaluation of cytotoxicity of nanoconjugates by MTT protocols

MTT tests were selected for evaluating the cytotoxicity of AIS@CMC and AIS@CMC_Cys nanoparticles functionalized with KLA and KLAR7 peptides. Briefly, all samples were added to individual wells at a final QD concentration of 3.5 nM and KLA and KLAR7 concentrations of 0.8 μM (bioconjugate with peptide:COO^-^ = 1:20) and 4 μM (bioconjugate with peptide:COO^-^ = 1:4) calculated from KLA amount added in the synthesis. All samples were incubated with U-87 MG and HEK 293T cells for 6 h and 24 h, and the percentages of cell viability and toxicity were calculated according to Eq. 1 and Eq. 2, respectively. Values of control (wells with cells and no samples) were considered as 100% of cell viability.

Cell viability = (Absorbance of sample and cells/Absorbance of control) x 100 % (1)

Toxicity = 100% - cell viability (2)

For dose-response curves, MTT assay was performed by incubating U-87 MG cells with AIS@CMC_KLA or AIS@CMC_Cys_KLA for 24 h. KLA peptide concentrations varied from 0.0003-3 μM based on KLA amount added in the synthesis and using samples with peptide:COO^-^ ratio equal to 1:20.

For evaluation of nanocarriers (AIS@CMC-DOX, AIS@CMC_Cys-DOX, AIS@CMC_KLA-DOX, and AIS@CMC_Cys_KLA-DOX and Free DOX), samples were incubated with U-87 MG and HEK 293T cells for 24 h. Nanocarriers were tested at a final concentration of 3.5 nM of QD, 4 μM of KLA (bioconjugate with peptide:COO^-^ = 1:4), and 4 µM of DOX. Both KLA and DOX concentrations were calculated considering the payloads added in the synthesis of nanoconjugates.

#### Confocal Laser Scanning Microscopy experiments

The evaluation of the cell-uptake of conjugates based on AIS fluorescence emission, of the mitochondrial signals in cells treated with bioconjugates and stained with MitoTracker™ and of DOX drug distribution in cells were performed by CLSM using Eclipse Ti confocal microscope (Nikon Instruments). U-87 MG cells were treated with the nanoconjugates and nanocarriers for different times of incubation (usually 15 min, 2 h, and 6 h). For analyzing mitochondria signals, cells were treated with MitoTracker™ Deep Red FM after being exposed to the nanoconjugates.

#### Evaluation of Reactive Oxygen Species (ROS) formation

For the evaluation of the formation of intracellular ROS, U-87 MG cells were treated with 2',7'-dichlorodihydrofluorescein diacetate (DCF-DA) fluorogenic probe and incubated with AIS@CMC, AIS@CMC_KLA, AIS@CMC_KLAR7, AIS@CMC_Cys, AIS@CMC_Cys_KLA, and AIS@CMC_Cys_KLAR7. After incubation times of 0 (control), 15 min, 30 min, and 60 min, the fluorescence intensity associated with the formation of the highly fluorescent 2',7'-dichlorofluorescein (DCF) was measured under λ_excitation_ = 485 nm and at λ_emission_ = 528 nm (Varioskan™ LUX multimode microplate reader, Thermo Scientific).

#### Evaluation of bioconjugate internalization using steady-state fluorescence

In addition to the analysis of cellular uptake of KLA-modified conjugates by CLSM, measurements of AIS emission following cell internalization of bioconjugates were performed by steady-state fluorescence spectroscopy (FluoroMax-Plus-CP, Horiba Scientific) following a previously published protocol 24. The data of PL intensity per cell *versus* Time were fitted to pseudo-first-order law and pseudo-second-order law aiming at comparing the cellular uptake kinetics of the samples 88.

## Figures and Tables

**Table 1:** Summary of sample identification and active species.

Sample identification	Fluorescent species	Cell-penetrating moiety	Cell death agent
AIS@CMC_Cys	AIS	Cys	NA
AIS@CMC_Cys_KLA	AIS	Cys	KLA
AIS@CMC_Cys_KLAR7	AIS	Cys and R7	KLA
AIS@CMC	AIS	NA	NA
AIS@CMC_KLA	AIS	NA	KLA
AIS@CMC_KLAR7	AIS	R7	KLA
AIS@CMC-DOX	AIS - green DOX - red	NA	DOX
AIS@CMC_KLA-DOX	AIS - green DOX - red	NA	KLA and DOX
AIS@CMC_Cys-DOX	AIS - green DOX - red	Cys	DOX
AIS@CMC_Cys_KLA-DOX	AIS - green DOX - red	Cys	KLA and DOX

NA = not available.

**Table 2:** *In silico* simulation of properties of KLA and KLAR7 peptides.

Peptide	Number of residues	Molar mass (g/mol)	Isoelectric point	Net charge at pH 7
KLA	13	1395.8	11.3	5
KLAR7	21	2617.3	12.9	13

**Table 3:** Hydrodynamic diameter (D_H_) and zeta potential (ZP) values of AIS@CMC-based nanostructures.

Sample	D_H_ (nm)/PdI	ZP (mV)
AIS@CMC	74 / 0.385	- 49 ± 3
AIS@CMC_KLA	141 / 0.336	- 41 ± 5
AIS@CMC_KLAR7	95 / 0.330	- 10 ± 1
AIS@CMC_Cys	66 / 0.247	- 18 ± 1
AIS@CMC_Cys_KLA	139 / 0.311	- 13 ± 1
AIS@CMC_Cys_KLAR7	99 / 0.370	- 23 ± 5

**Table 4:** Average lifetimes (τ_av_) of AIS@CMC-based fluorescent nanostructures.

Sample	τ_av_ for λ_emission_ = 625 nm (ns)
AIS@CMC	407
AIS@CMC_KLA	182
AIS@CMC_KLAR7	225
AIS@CMC_Cys	312
AIS@CMC_Cys_KLA	211
AIS@CMC_Cys_KLAR7	226
