# Supplementary material for: Nanotheranostics through Mitochondria-targeted Delivery with Fluorescent Peptidomimetic Nanohybrids for Apoptosis Induction of Brain Cancer Cells
Source: Nanotheranostics. 2021 Feb 8;5(2):213–39. doi: 10.7150/ntno.54491 (PMC7893535; doi:10.7150/ntno.54491)
Supplement: Supplementary file 1 — Supplementary figures and tables. [file ntnov05p0213s1.pdf]

Supplementary Material

Nanotheranostics through Mitochondria-targeted Delivery with Fluorescent Peptidomimetic Nanohybrids for Apoptosis Induction of Brain Cancer Cells

Isadora C. Carvalho[1], Alexandra A. P. Mansur[1], Sandhra M. Carvalho[1], Herman S. Mansur[1,*]

[1]*Center of Nanoscience, Nanotechnology, and Innovation - CeNano²I, Department of Metallurgical and Materials Engineering, Federal University of Minas Gerais - UFMG, Av. Antônio Carlos, 6627 – Belo Horizonte/MG, Brazil.*

**E-mail: hmansur@demet.ufmg.br*

Results and Discussion

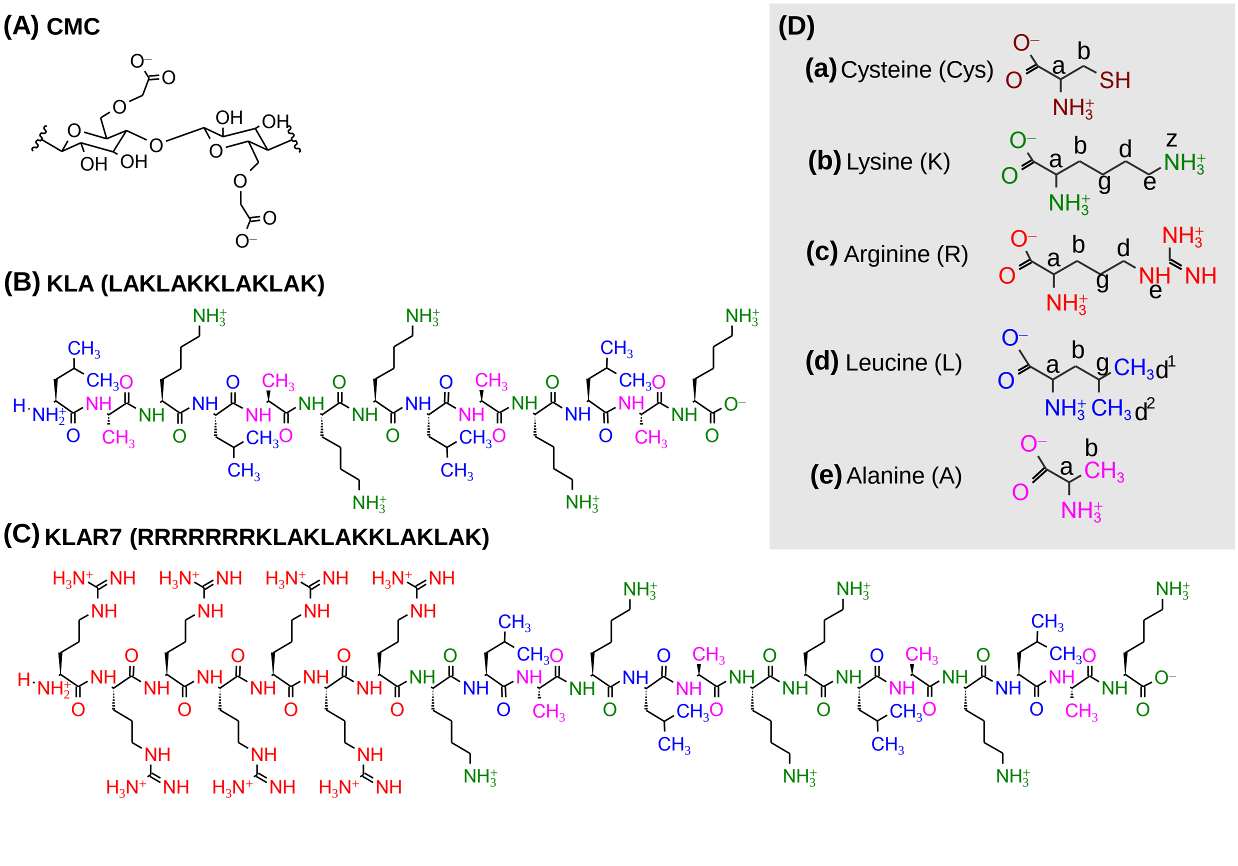

Figure S1. Chemical structures of (A) Carboxymethylcellulose (CMC), (B) KLA, (C) KLAR7, and (D) (a) Cysteine, (b) Lysine, (c) Arginine, (d) Leucine, and (e) Alanine.

*To whom correspondence should be addressed: Federal University of Minas Gerais, Av. Antônio Carlos, 6627 – Escola de Engenharia, Bloco 2 – Sala 2233, 31.270-901, Belo Horizonte/MG, Brazil; Phone: +55-31-34091843; E-mail: hmansur@demet.ufmg.br (H. Mansur)

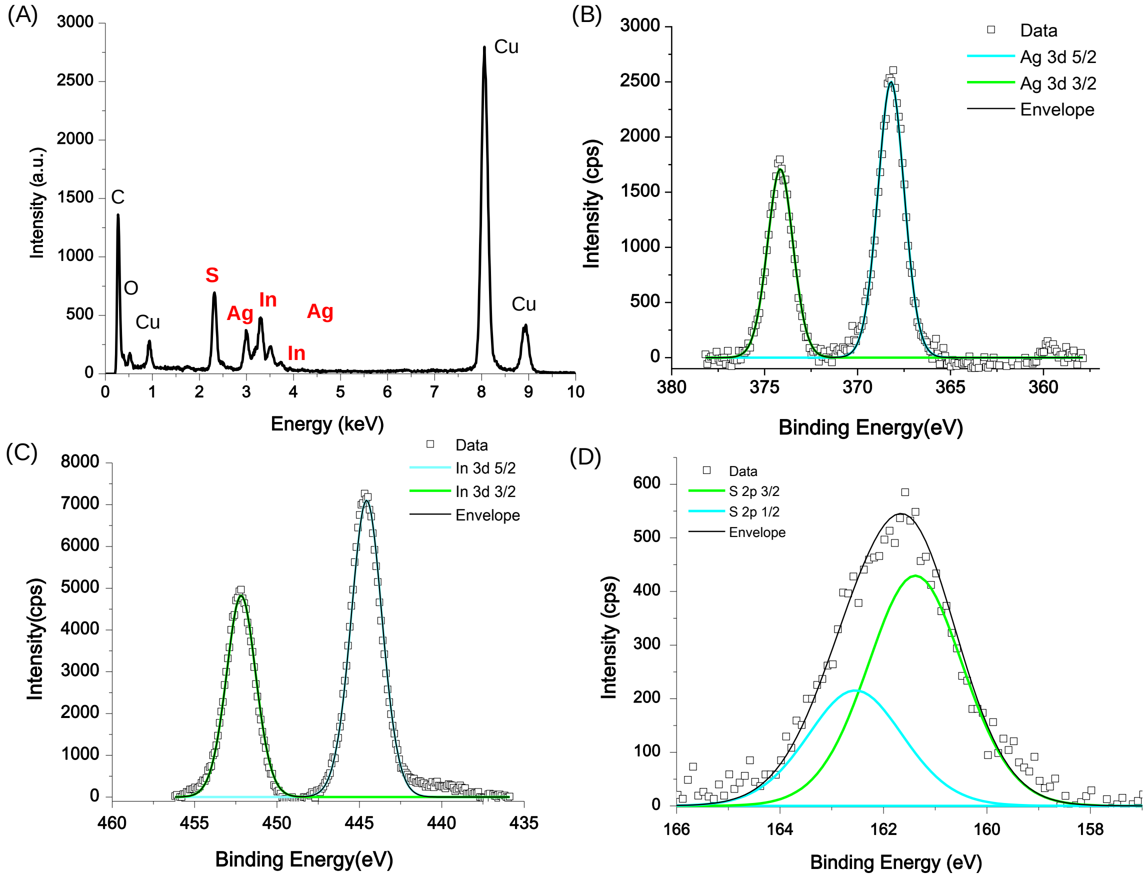

Figure S2. (A) EDX spectra of AIS@CMC. (B) Ag 3d XPS spectra of AIS@CMC. (C) In 3d XPS spectra of AIS@CMC. (D) S 2p XPS spectra of AIS@CMC.

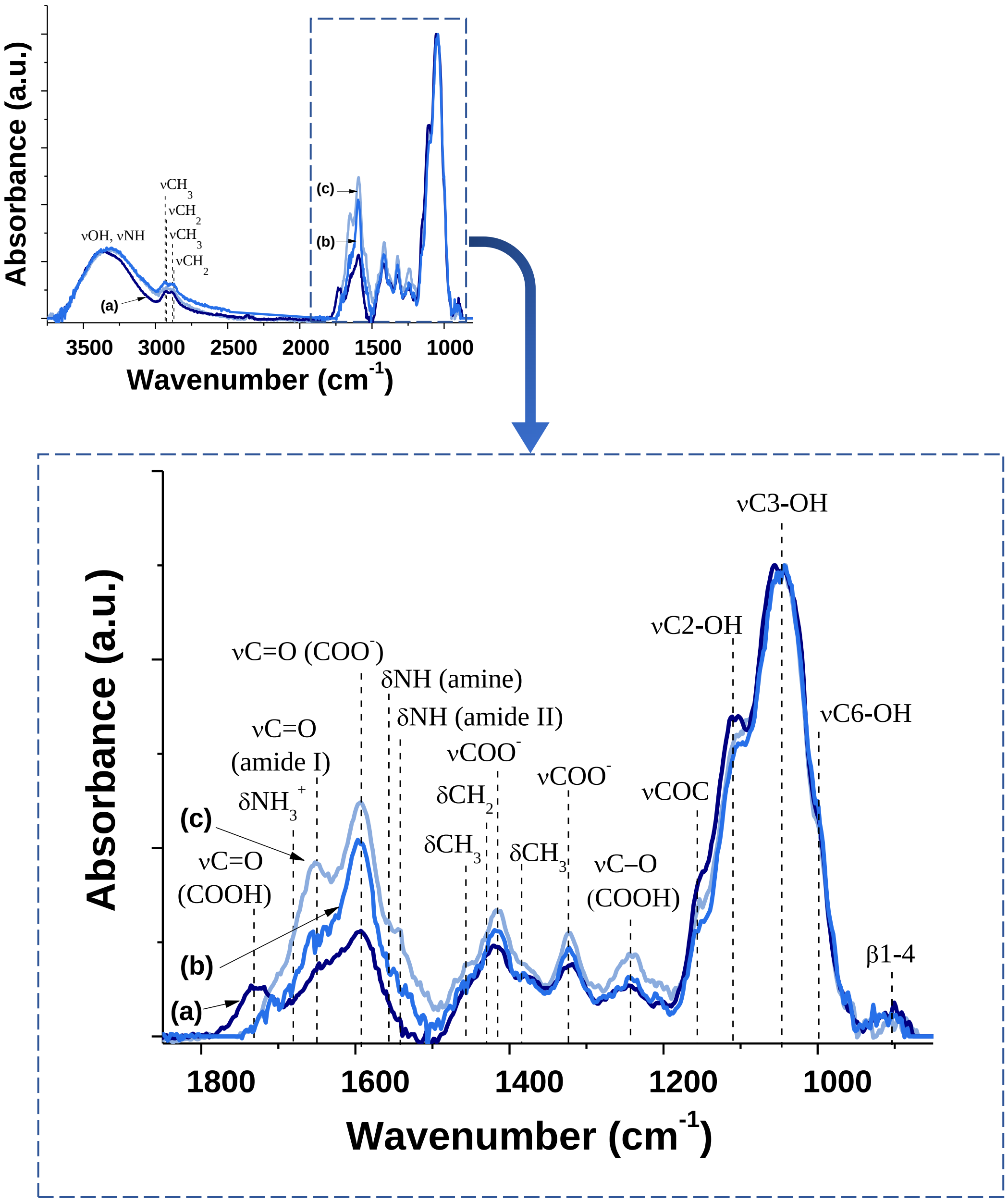

Figure S3. FTIR spectra of nanoconjugates: (a) AIS@CMC, (b) AIS@CMC_ KLA, and (c) AIS@CMC_KLAR7.

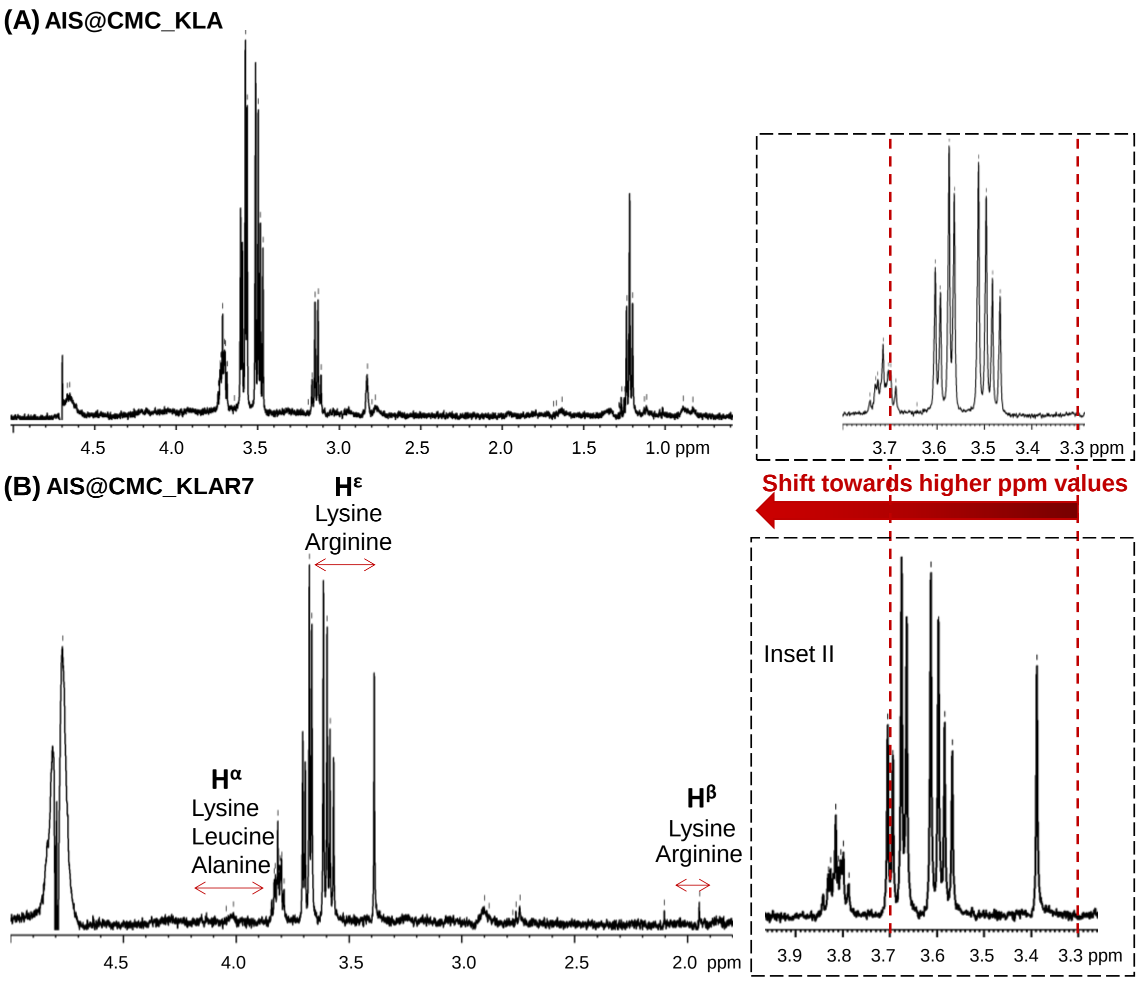

Figure S4. ^{1}H NMR spectra of (A) AIS@CMC_KLA, and (B) AIS@CMC_KLAR7.

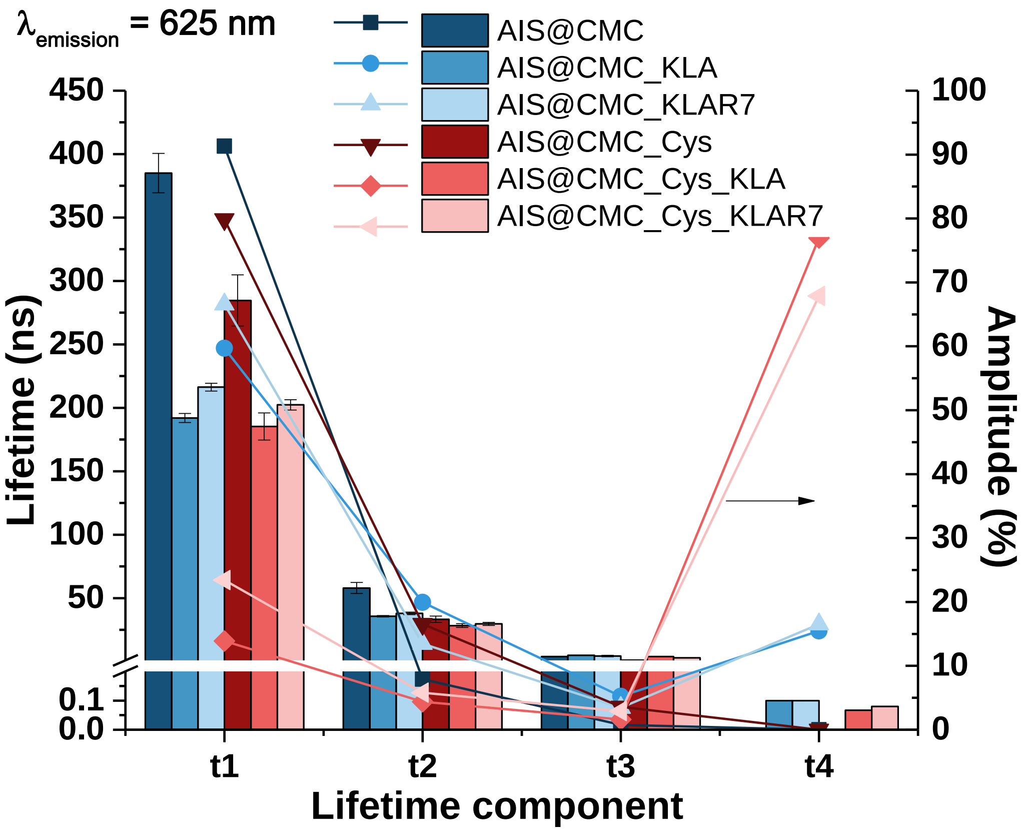

Figure S5. Decay times (τ_i) and amplitude ratios (A_i) of the PL emission at 625 nm.

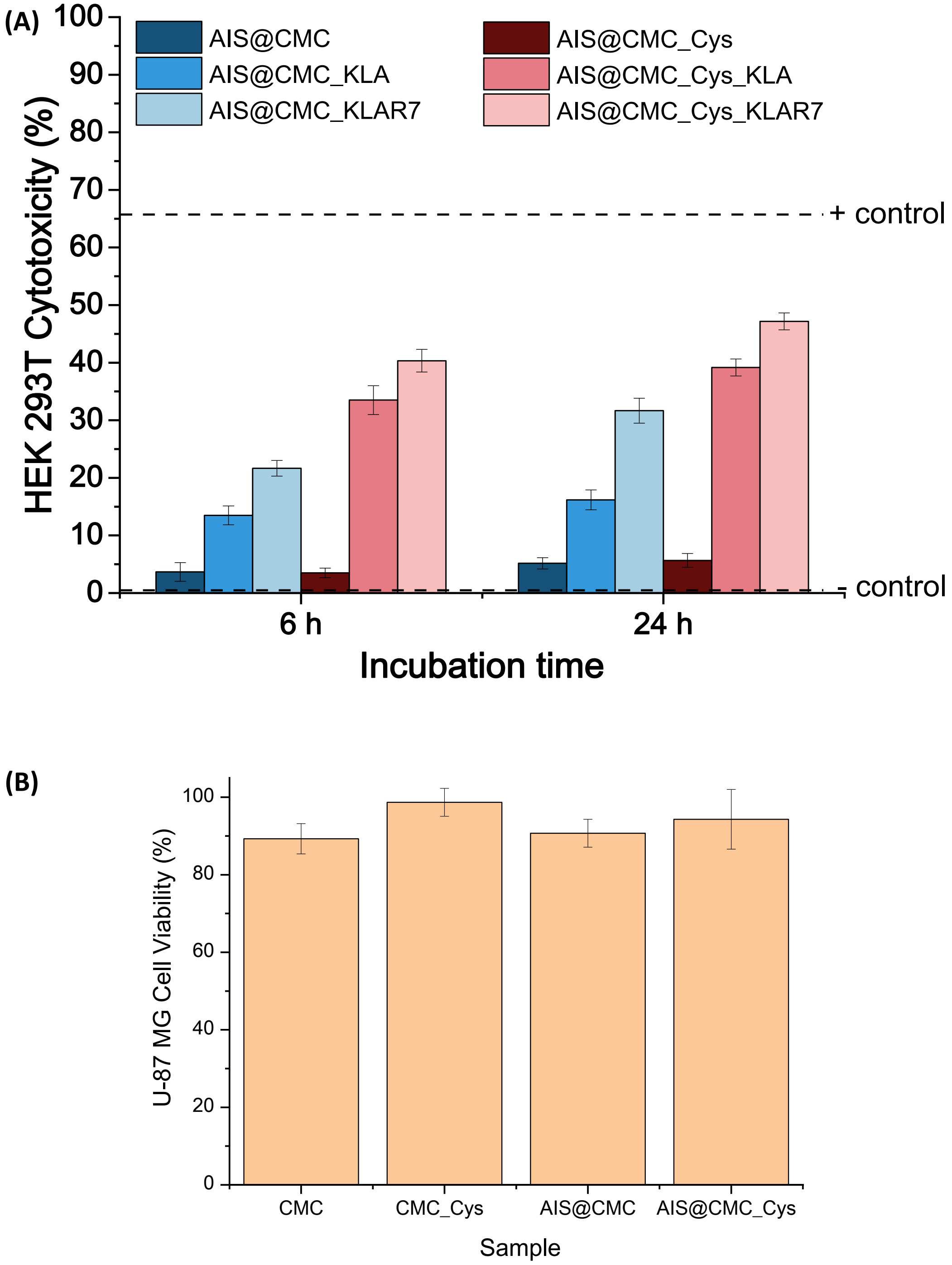

Figure S6. (A) Cytotoxicity responses of nanoconjugates after 6 h and 24 h of incubation with HEK 293T cell line ([peptide] = 0.8 µM). (B) Cell viability results of CMC polymer, CMC_Cys thiomer, AIS@CMC, and AIS@CMC_Cys after 6 h of incubation with U-87 MG cells.

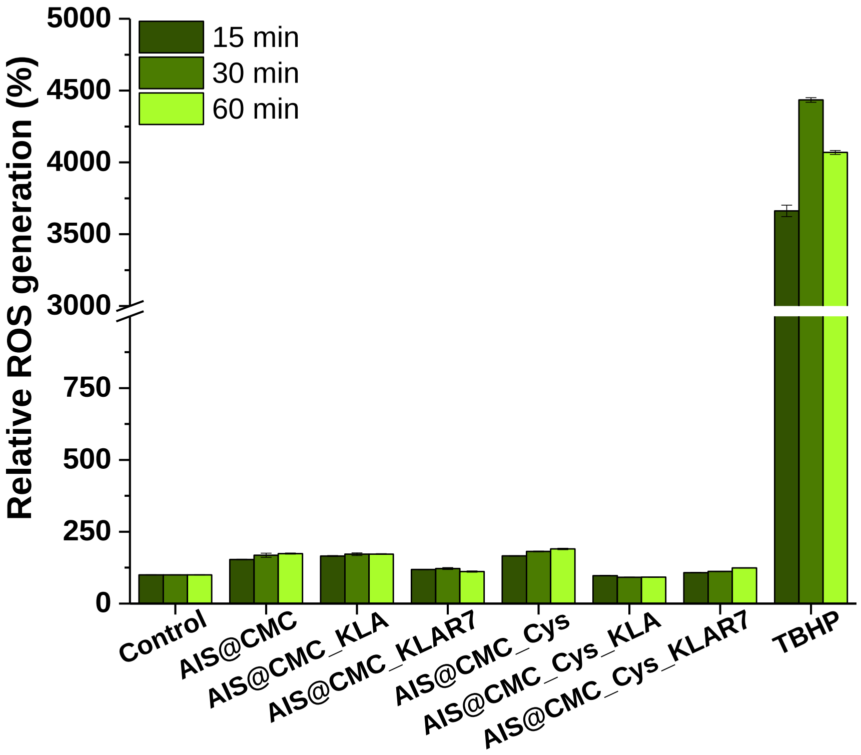

Figure S7. Accumulation of intracellular ROS in U-87 MG cells induced by nanoconjugates after incubation times of 15 min, 30 min, and 60 min (mean ± SD; n = 8).

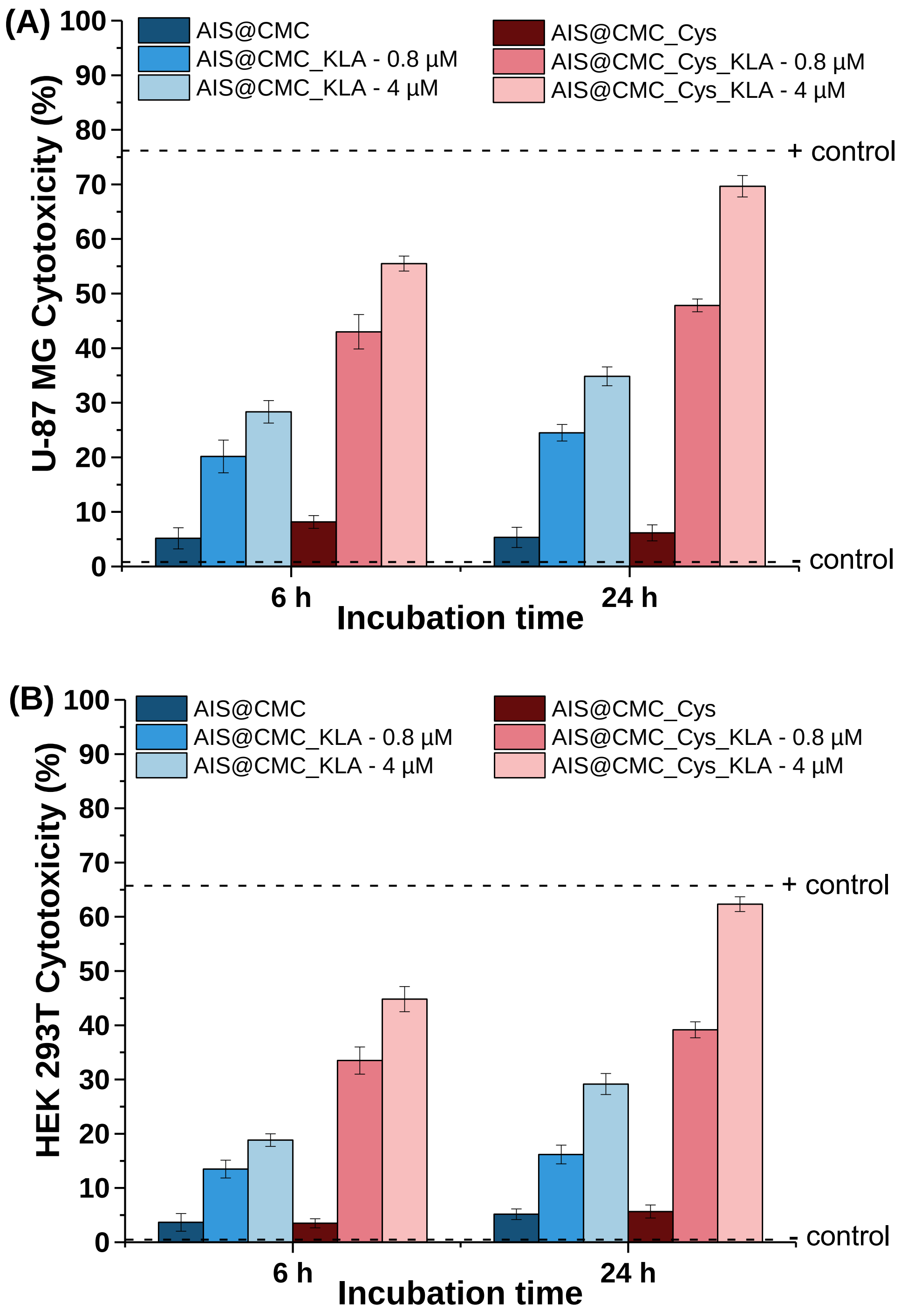

Figure S8. Comparison of cytotoxicity values for U-87 MG (A) and HEK 293T (B) cells after incubation for 6 h and 24 h with AIS@CMC_KLA and AIS@CMC_Cys_KLA with peptide concentration of 0.8 µM and 4 µM.

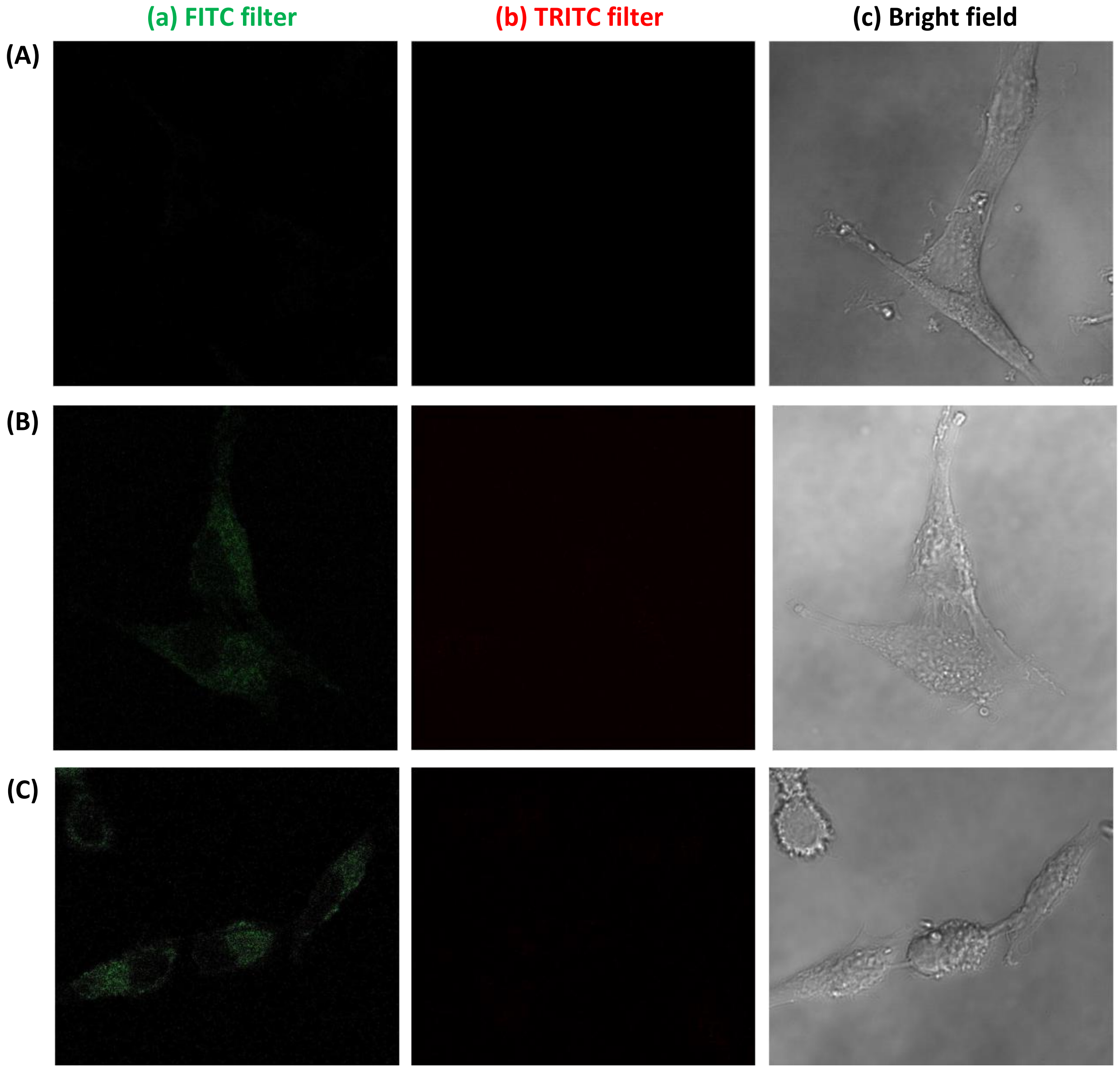

Figure S9. CLSM images of (A) control sample (autofluorescence, cells + medium without nanoconjugates), (B) AIS@CMC and derivatives, and (C) AIS@CMC_Cys and derivatives using (a) FITC and (b) TRITC filters and (c) bright field images.

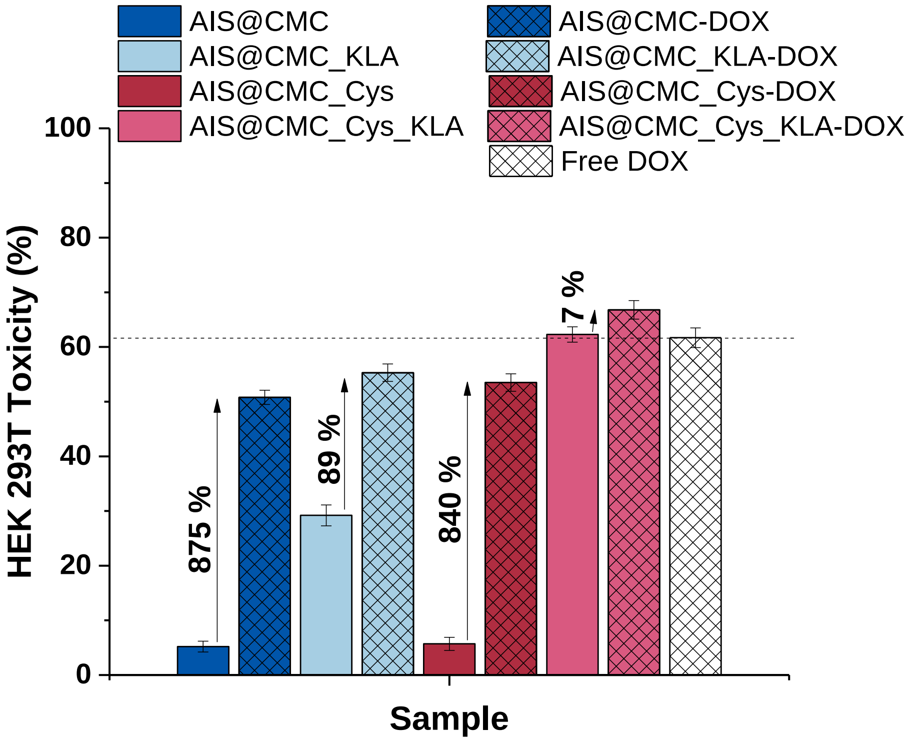

Figure S10. MTT results for conjugates and nanocarriers after incubation for 24 h with HEK 293T cell line ([DOX] = 4 µM, and [KLA] = 4 µM).

Methods

Materials

Ethanolamine hydrochloride (ETA, $H_2NCH_2CH_2OH{\cdot}HCl$, ≥ 99.0 %), sodium sulfide nonahydrate ($Na_2S{\cdot}9H_2O$, ≥ 98 %), 1-ethyl-3-[3- (dimethylamine)propyl]carbodiimide hydrochloride (EDC, ≥ 98 %), N-hydroxysulfosuccinimide sodium salt (sulfo-NHS, $C_4H_4NNaO_6S$, ≥ 98 %), sodium phosphate dibasic (Na_2HPO_4, ≥ 99.0 %), potassium phosphate monobasic (KH_2PO_4, ≥ 99.0 %), potassium chloride (KCl, ≥ 99.0 %), sodium chloride (NaCl, ≥ 99.0 %), 5,5′-dithiobis(2-nitrobenzoic acid) (DTNB, Ellman's reagent, 99 %), Triton™ X-100, sodium dodecyl sulfate (SDS, ≥ 99.0 %), 2-(N-morpholino)-ethanesulfonic acid (MES, > 99 %, with low moisture content), 3-(4,5- dimethylthiazol-2yl) 2,5-diphenyltetrazolium bromide (MTT, > 98 %), indium nitrate ($In(NO_3)_3{\cdot}xH_2O$, In ≥ 28.5 % wt.), hydrochloric acid (HCl, 37 %), and paraformaldehyde (> 95 %) were purchased from Sigma-Aldrich (USA). Ethylenediaminetetraacetic acid (EDTA, $C_{10}H_{16}N_2O_8$, ≥ 99.9 %), fetal bovine serum (FBS), streptomycin sulfate, amphotericin-b, penicillin G sodium, and Dulbecco's Modified Eagle Medium (DMEM) were supplied by Gibco BRL (USA). Silver nitrate ($AgNO_3$, 99.9 %) was purchased from Synth (Brazil). Hydromount™ was purchased from Fisher Scientific Ltd. MitoTracker® Deep Red FM was supplied by Thermo Fisher Scientific (USA). Unless otherwise specified, all chemicals, reagents, and precursors were used as supplied without any additional purification procedure. All solutions used deionized water with the minimum resistivity of 18 MΩ cm (DI water, Millipore Simplicity™), and the processes were performed at room temperature (RT, 23 ± 2 ºC).

Synthesis of AIS@CMC colloidal solution

The synthesis of ternary quantum dots of Ag-In-S (AIS QDs) using carboxymethylcellulose (CMC) as both stabilizing ligand and functionalization agent was performed as follows. CMC polymer solution (0.04 wt. %) was prepared in DI water and homogenized by magnetic stirring. Then, 0.33 mL of $AgNO_3$ (10 mM) was added to 48 mL of the polymer solution and vigorously stirred for 1 min. Following, 1.33 mL of $In(NO_3)_3{\cdot}xH_2O$ (10 mM) were pipetted into the reaction flask and stirred for 1 min ([Ag:In] molar ratio = 1:4). Finally, 2.16 mL of $Na_2S{\cdot}9H_2O$ (10 mM) was rapidly poured into the reaction flask and vigorously stirred for 10 min. The colloidal solution was submitted to thermal treatment for annealing/growth of $AgInS_2$ QDs by heating at

100 ± 5 °C for 10 min. The resulting $AgInS_2$ colloidal dispersion was referred to as AIS@CMC, and it was dialyzed using a cellulose membrane with cut-off (MWCO) of 12 kDa (Sigma-Aldrich) for 24 h against distilled water with two changes of the dialysate for eliminating unreacted precursors. The nanoconjugate solution was stored in plastic flasks at RT.

Functionalization of AIS@CMC QD with L-cysteine

EDC was used as a zero-length crosslinker for CMC functionalization with Cys residues by amide bond formation. In brief, 50 mL of AIS@CMC suspension previously prepared was dried in an oven using hot air at 40 ± 1 °C to achieve a volume of 23.5 mL by solvent evaporation. Then, 23.5 mL of MES buffer 0.5 M (pH 5.5 ± 0.1) was added to the QDs suspension and stirred until homogenization, and 1 mL of EDC solution in MES (0.25 M, pH 5.5 ± 0.1, 2-fold related to COO^- groups of AIS@CMC) was dropped into the reaction flask and stirred for 15 min for activating the carboxylate groups of the polymer. In the sequence, 2.0 mL of MES solution (0.25 M, pH 5.5) containing 2 wt. % of L-cysteine hydrochloride (2-fold related to COO^- groups of AIS@CMC) was poured into the AIS@CMC/EDC suspension, and the system was incubated for 2 h under magnetic stirring with the reaction flask covered to minimize light exposure. The EDC-mediated crosslinking reaction was quenched by adding 1.5-fold of ETA (related to EDC). The resulting AIS@CMC_Cys suspension was dialyzed (dialysis tubing cellulose membrane, 12-14 kDa cut-off, Sigma-Aldrich) for 48 h and dried using hot air at 40 ± 1 °C to achieve a volume of 50 mL by solvent evaporation.

Characterization of quantum dots, thiomers, peptidomimetic nanoassemblies, and nanocarriers

Ultraviolet-visible (UV-Vis) and photoluminescence (PL) spectroscopies were applied for accessing the absorption and emission properties of all nanoconjugates, respectively. UV−vis spectra were acquired at 190 nm < λ < 700 nm in transmission mode using the Lambda EZ- 210 spectrometer (PerkinElmer Inc., USA). PL emission spectra were collected using FluoroMax-Plus - CP (Horiba Scientific, Japan) with $\lambda_{excitation}$ = 350 nm and $\lambda_{emission}$ = 400-800 nm. Quantum yield (QY) parameter was estimated according to the comparative procedure using Rhodamine 6G (Sigma, USA) in ethanol as the standard at λ_{exc} = 488 nm [1]. The time-correlated single-

photon counting setup from Horiba (DeltaDiode - pulsed laser peak at 375 ± 10 nm) was used for collecting the photoluminescence lifetime decay at $\lambda_{emission}$ = 625 nm. The fluorescence decay curves were fitted by the multiexponential analysis using Eq. S1, and the average lifetime decay, τ_{av}, was calculated according to the Eq. S2.

$$I(t) = \sum_{i=1}^{n} A_i \, e^{\left(\frac{-t}{\tau_i}\right)} \qquad \text{(S1)}$$

$$\tau_{av} = \frac{\sum_{i=1}^{n} A_i \times \tau_i^2}{\sum_{i=1}^{n} A_i \times \tau_i} \qquad \text{(S2)}$$

where *Ai* is the relative amplitude of the decay components associated with the PL lifetimes τ_i.

The morphological characterization of QD nanostructures was based on images obtained using Tecnai G2- 20-FEI (FEI Company, USA) transmission electron microscope (TEM) coupled with energy-dispersive x-ray spectroscopy (EDX, EDAX detector) for elemental chemical analysis at the accelerating voltage of 200 kV. For preparing samples, AIS@CMC QD suspension was centrifuged and washed with DI water using Amicon® Ultra Centrifuge Filter (30 kDa cut-off cellulose membrane, Sigma-Aldrich, 4 cycles × 5 min, 12,000 rpm). The retained material was resuspended in 450 μL of DI water, dropped onto carbon-coated copper grids (Electron Microscopy Sciences, USA) and dried at room temperature. The size and size-distribution data of quantum dots were assessed based on the TEM images with at least 100 randomly selected nanoparticles using *DigitalMicrograph*™ image processing software (Gatan, Inc.), and the PdI was calculated according to Eq. S3 [2].

$$PdI = \left(\frac{\sigma}{d}\right)^2 \qquad \text{(S3)}$$

where σ is the standard deviation, and *d* is the mean particle diameter.

Also, atomic force microscopy (AFM) images of the AIS@CMC QDs were obtained using XE-70 (Parker Systems Inc., USA). The instrument was operated in non-contact tapping mode (frequency = 325 kHz). The scanning rate was 1.0 Hz, and the images were acquired with a 528 × 528 pixel resolution. The samples were prepared as described for TEM experiments; however, the retained material was dropped onto plastic molds and dried in a hot-air oven at 40 ± 1 °C for 24 h forming films that were fixed onto the AFM sample holder.

X-ray photoelectron spectroscopy (XPS) analysis was performed using Mg-Kα as the excitation source (Amicus spectrometer, Kratos Analytical, Japan). All peak positions were corrected based on C 1s binding energy (285 eV). For sample preparation, a concentrated colloidal medium of QD was dropped onto a plastic mold and dried in an oven (see AFM sample preparation). In the sequence, samples were dehydrated in absolute ethanol (3 immersions of 30 s each) and dried in a conventional vacuum desiccator at room temperature for 2 h.

Fourier Transformed Infrared Spectroscopy (FTIR) spectra were obtained using attenuated total reflectance (ATR, 4000–850 cm^{-1} using 32 scans and a 4 cm^{-1} resolution - Nicolet 6700, Thermo-Fischer, USA) with background subtraction. Films of conjugates were prepared as described for AFM analysis. All FTIR experiments were conducted in duplicate (n = 2) unless expressly noted.

^{1}H-NMR (proton nuclear resonance spectroscopy) spectra were recorded by Avance™III DH NanoBay 400 MHz, Bruker (64 scans), and analyzed by TopSpin 3.1 software.

Analyses of zeta potential and DLS (dynamic light scattering) were carried at RT using the ZetaPlus instrument with a minimum of ten replicates (Brookhaven Instruments Corporation, UK, 35 mW red diode laser light, wavelength λ = 660 nm).

For estimating the degree of insertion of Cys residues in AIS_CMC QDs, the Ellman's method was applied following a previous publish protocol with slight modifications [3]. Firstly, an Ellman's Reagent Solution was prepared by dissolving 8 mg of DTNB in 2 mL of a 0.1 M sodium phosphate buffer (at pH = 8.0 ± 0.1) containing 1 mM of EDTA. Then, a stock solution of Cys in the same buffer was prepared at a final concentration of 4.8 mM. For plotting the working curve (Figure S11), a set of seven test tubes were prepared by mixing 50 µL of Ellman's Reagent Solution, 2.5 mL of sodium phosphate buffer containing 1 mM of EDTA, and 250 µL of Cys solutions with concentrations varying from 0-1.5 mM in sodium phosphate buffer containing 1 mM of EDTA.

For preparing the sample set, test tubes were prepared by mixing 250 µL of the AIS_CMC_Cys solution with 50 µL of Ellman's Reagent Solution and 2.5 mL of sodium phosphate buffer containing 1 mM of EDTA. All test tubes were left reacting for 15 min, and the absorbance at 412 nm was measured for each sample (Lambda EZ- 210, PerkinElmer Inc., USA). Thiol concentrations were calculated basing on the Beer-Lambert law (Eq. S4).

$$\varepsilon = \frac{A}{l.c} \qquad \text{(S4)}$$

where ε is the molar coefficient at 412 nm, A is the absorbance at 412 nm, l is the path length (cm), and c is the molar concentration.

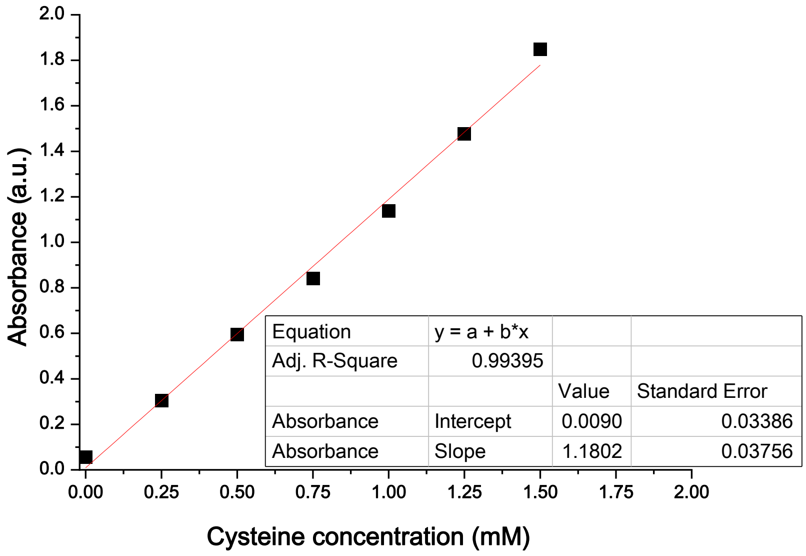

Figure S11. Working curve for the determination of thiol concentration by Ellman's Method. The filled squares represent the acquired data, and the red line the linear regression for experimental data.

Biological experiments

All biological tests were conducted according to ISO 10993-5:2009/(R)2014 (Biological Evaluation of medical devices: tests for in vitro cytotoxicity. U-87 MG (passage 25) and HEK 293T (passage 19) cells were cultured in DMEM (Dulbecco's Modified Eagle Medium) with 10 % FBS (fetal bovine serum), streptomycin sulfate (10 mg mL^{-1}), penicillin G sodium (10 units mL^{-1}), and amphotericin-b (0.025 mg mL^{-1}) in a humidified atmosphere of 5 % CO_2 at 37 ˚C.

Evaluation of cytotoxicity in vitro by MTT cell viability assay. MTT experiments were performed to evaluate the cytotoxicity of conjugates and free DOX after incubation with cell lines for different times (6 h and 24 h) and peptides concentration (0.8 µM and 4 µM, based on KLA loading), depending on the experiment. All the cells were plated (1×10^4 cells/well) in 96-

well microplates. Each cell population was synchronized in serum-free media for 24 h. Next, the media volume was aspirated and replaced with a DMEM medium containing 10% FBS. Then, samples were added to individual wells dispersed/dissolved with DMEM medium containing 10% FBS. After 6 h and/or 24 h, all media were suctioned and replaced with the culture medium containing serum to each well (60 µL). MTT solution (5 mg mL^{-1}) was added to each well and incubated for 4 h. Then, SDS solution/4% HCl (40 µL) was placed in every well and incubated for 16 h in an oven (37 ˚C, 5% CO_2). Then, 100 µL was removed from each well and transferred to a 96-well microplate, and the absorbance was measured (iMarkTM Microplate Absorbance Reader, Bio-Rad©, USA, with wavelength filter at λ=595 nm). The control samples were planned as follows: control group ("Control", cell culture with DMEM and 10% FBS); positive control ("+ Control", cell culture with DMEM, 10% FBS, and 1.0% v/v Triton™ X-100); and negative control ("- Control", cell culture with DMEM, 10% FBS and *"chips"* of sterile polypropylene Eppendorf®, 1 mg mL^{-1}, Eppendorf, Germany).

Statistical significance was tested through One-way ANOVA followed by Bonferroni's method using the OriginPro® 2020 software (OriginLab®) with a significance level (α) of 0.05. At p-value (or probability value) < 0.05, the result was considered statistically significant. The experiments were performed using at least in sextuplicate ($n \geq 6$).

Evaluation of Reactive Oxygen Species (ROS) formation. U-87 MG cells (1×10^4 cells/well on 96-well plates) were incubated with 100 µL of 2',7'-dichlorodihydrofluorescein diacetate (DCF-DA) solution at 100 µM (diluted in culture medium) for 40 min in an oven at 37 ˚C and at the atmosphere of 5 % CO_2. After this period, the probe was removed, and the cells were exposed to AIS@CMC, AIS@CMC_KLA, AIS@CMC_KLAR7, AIS@CMC_Cys, AIS@CMC_Cys_KLA, and AIS@CMC_Cys_KLAR7 nanoconjugates. For each sample, 40 µL of nanoconjugate colloidal solution were mixed with 1.56 mL of culture medium (final concentration of 0.8 µM of peptide and 3.5 nM of QD), and 100 µL were added to each well. After incubation times of 0 (control), 15 min, 30 min, and 60 min, the fluorescence intensity at each well was measured under $\lambda_{excitation}$ = 485 nm and at $\lambda_{emission}$ = 528 nm using filters on Varioskan™ LUX multimode microplate reader (Thermo Scientific, USA). For negative control, cells were incubated with DCF-DA and, for positive control, cells were treated with tert-butyl hydrogen peroxide (TBHP, 5.0 mM in water). The result was showed as a percentage of

fluorescence intensity considering negative control wells as reference (100 %). Data and results were expressed as means and standard deviations (SD) of eight replicates (n = 8).

Cellular uptake of QD nanoconjugates by laser scanning confocal microscopy. The cells were plated at 5×10^5 cells / well in a 6-well plate, incubated for 24 h (oven at 37 ˚C, 5% CO_2), and synchronized for 24 h. Then, samples (1:1, v/v) with the medium solution (DMEM solution with 10 % FBS) were added to the cells and incubated in 5 % CO_2 at 37 ˚C for 15 min, 2 h, and 6 h followed by washing with PBS solution. For the evaluation of mitochondria signals, cells were additionally stained with MitoTracker™ Deep Red FM according to the manufacturer's protocol. Then, the cells were fixed using a paraformaldehyde solution (4.0 % in PBS) for 30 min, washed three times with PBS, and coverslips were mounted with Hydromount®. Digital images were taken using a confocal microscope (Eclipse Ti, Nikon Instruments, USA) using the oil immersion objective (63× Plan-Apo/1.4 NA). Excitation was performed at $\lambda_{excitation}$ = 488 nm (fluorescein isothiocyanate, FITC) with emission collected $\lambda_{emission}$ = 506-550 nm, for typical nanoconjugate green signals, and at $\lambda_{excitation}$ = 543 nm (tetramethylrhodamine, TRITC) emission collected at $\lambda_{emission}$ = 545−645 nm for red-emitting DOX or MitoTracker™ Deep Red FM. Two-color confocal fluorescence images were recorded separately in the correspondent channel and merged afterward.

For the reference control, cells were incubated using only with the original medium with 10 % FBS (autofluorescence). Then, measurements of mean fluorescence intensity (MFI) were performed based on public domain image processing software (ImageJ software, version 1.50+).

Cellular uptake of QD nanoconjugates by steady-state fluorescence. Glioblastoma (U-87 MG) cells were plated in 6-well plates (5×10^4 cells/well), synchronized in serum-free media for 24 h, and incubated for 24 h in an oven (temperature 37 ˚C and atmosphere of 5 % CO_2). AIS@CMC_KLA and AIS@CMC_Cys_KLA colloidal solutions were poured into culture media at 1:1 (v/v) and incubated for 1 min, 5 min, 15 min, 30 min, and 60 min. The U-87 MG cells were treated only with DMEN supplemented with 10 % FBS for reference control. After each treatment time, cells were washed with PBS, trypsinized for 7 min (250 μL of 0.2 % trypsin), collected, centrifuged (5 min at 1400 rpm Hettich Mikro 200R), and finally resuspended in 200 μL of PBS. Then, cell concentration was calculated by counting the number of cells in a

Neubauer Chamber using a microscope (CH30, Olympus Corporation, Japan). The PL steady-state emission spectra of the cell-containing solutions were collected under $\lambda_{excitation}$ = 360 nm (slit = 5 nm), and results were expressed by the ratio of PL intensity per cell. As a control, the autofluorescence of nanoconjugate-free suspended cells was measured under the same conditions.

Plots of the data of PL intensity per cell *versus* Time were fitted to pseudo-first-order law (Eq. S5) and pseudo-second-order law and its linear form (Eq. S6 and Eq. S7, respectively) aiming at semi-quantitatively comparing the uptake kinetics of the samples [4].

$$PL\ (t) = PL_{eq}(1 - e^{-k_1 t}) \qquad \text{(S5)}$$

$$PL\ (t) = PL_{eq}\ \frac{k_2 \times PL_{eq} \times t}{1 + (k_2 \times PL_{eq} \times t)} \qquad \text{(S6)}$$

$$\frac{t}{PL} = \frac{1}{k_2 PL_{eq}^2} + \left(\frac{1}{PL_{eq}} \times t\right) \qquad \text{(S7)}$$

Where k_1 is assigned to the pseudo-first-order rate constant (min^{-1}), k_2 is the pseudo-second-order rate constant ($cps^{-1}.min^{-1}$), and PL_{eq} and $PL\ (t)$ are the photoluminescence intensity (cps) at equilibrium and at time t (min), respectively.

References

1. Brouwer AM. Standards for photoluminescence quantum yield measurements in solution. Pure Appl Chem. 2011; 83: 2213-2228.
2. Clayton KN, Salameh JW, Wereley ST, Kinzer-Ursem TL. Physical characterization of nanoparticle size and surface modification using particle scattering diffusometry. Biomicrofluidics. 2016; 10: 054107.
3. Carvalho IC, Medeiros Borsagli FGL, Mansur AAP, Caldeira CL, Haas DJ, Lage AP, et al. 3D sponges of chemically functionalized chitosan for potential environmental pollution remediation: biosorbents for anionic dye adsorption and ‘antibiotic-free’ antibacterial activity. Environ Technol. 2019; November: 1–21.
4. Mäger I, Eiríksdóttir E, Langel K, Andaloussi SE, Langel Ü. Assessing the uptake kinetics and internalization mechanisms of cell-penetrating peptides using a quenched fluorescence assay. Biochim Biophys Acta Biomembr. 2010; 1798: 338–343.
